# Achieving record birefringence in metal-free semiorganic sulfate crystals *via* tetrahedral anionic group substitution

**DOI:** 10.1039/d6sc05559a

**Published:** 2026-08-17

**Authors:** Yan-Zhen Xie, Hai-Yan Jiang, Yi Zhang, Hui-Ping Xiao, Qing-Yan Liu, Yu-Ling Wang

**Affiliations:** a Key Laboratory of Porous Functional Materials of Jiangxi Province, Key Lab of Fluorine and Silicon for Energy Materials and Chemistry of Ministry of Education, College of Chemistry and Materials, Jiangxi Normal University Nanchang 330022 Jiangxi P. R. China qyliuchem@jxnu.edu.cn; b Department of Ecological and Resources Engineering, Fujian Key Laboratory of Eco-industrial Green Technology, Wuyi University Wuyishan 354300 Fujian P. R. China

## Abstract

Birefringent crystals with the ability to precisely modulate polarized light are indispensable optical devices in modern optics and optoelectronics. However, achieving high birefringence in such crystals remains a challenge. Integration of the π-conjugated 2-amino-5-methylpyridine (AMPy), which has large polarizability anisotropy, with non-π-conjugated tetrahedral groups produces two birefringent crystals, AMPy·PO_4_ and AMPy·SO_4_. AMPy·PO_4_ has a three-dimensional hydrogen-bonding structure, whereas AMPy·SO_4_ exhibits a two-dimensional hydrogen-bonded layer. Remarkably, upon substituting phosphate with sulfate, the experimental birefringence increases 3.8-fold, yielding a giant birefringence of 0.506 at 550 nm coupled with broad optical transparency and establishing AMPy·SO_4_ as a new benchmark for metal-free sulfate crystals. Structural and computational investigations reveal that the outstanding birefringence of AMPy·SO_4_ mainly originates from two key factors: the formation of a layered structure where tetrahedral sulfate anions modulate the arrangement of optically anisotropic AMPy cations to achieve nearly parallel alignment *via* hydrogen bonding, and the dense stacking of planar π-conjugated AMPy moieties. This work not only offers a universal strategy for designing semiorganic birefringent crystals *via* synergistically combining π-conjugated groups and non-π-conjugated rigid tetrahedral moieties but also pushes the birefringence limit of metal-free sulfate crystals to a new level.

## Abstract 2

Replacing phosphate with sulfate in the π‑conjugated 2‑amino‑5‑methylpyridine system boosts birefringence by optimizing the alignment and increasing the density of π‑conjugated groups, achieving record‑high values among metal‑free sulfate crystals.

## Graphical abstract

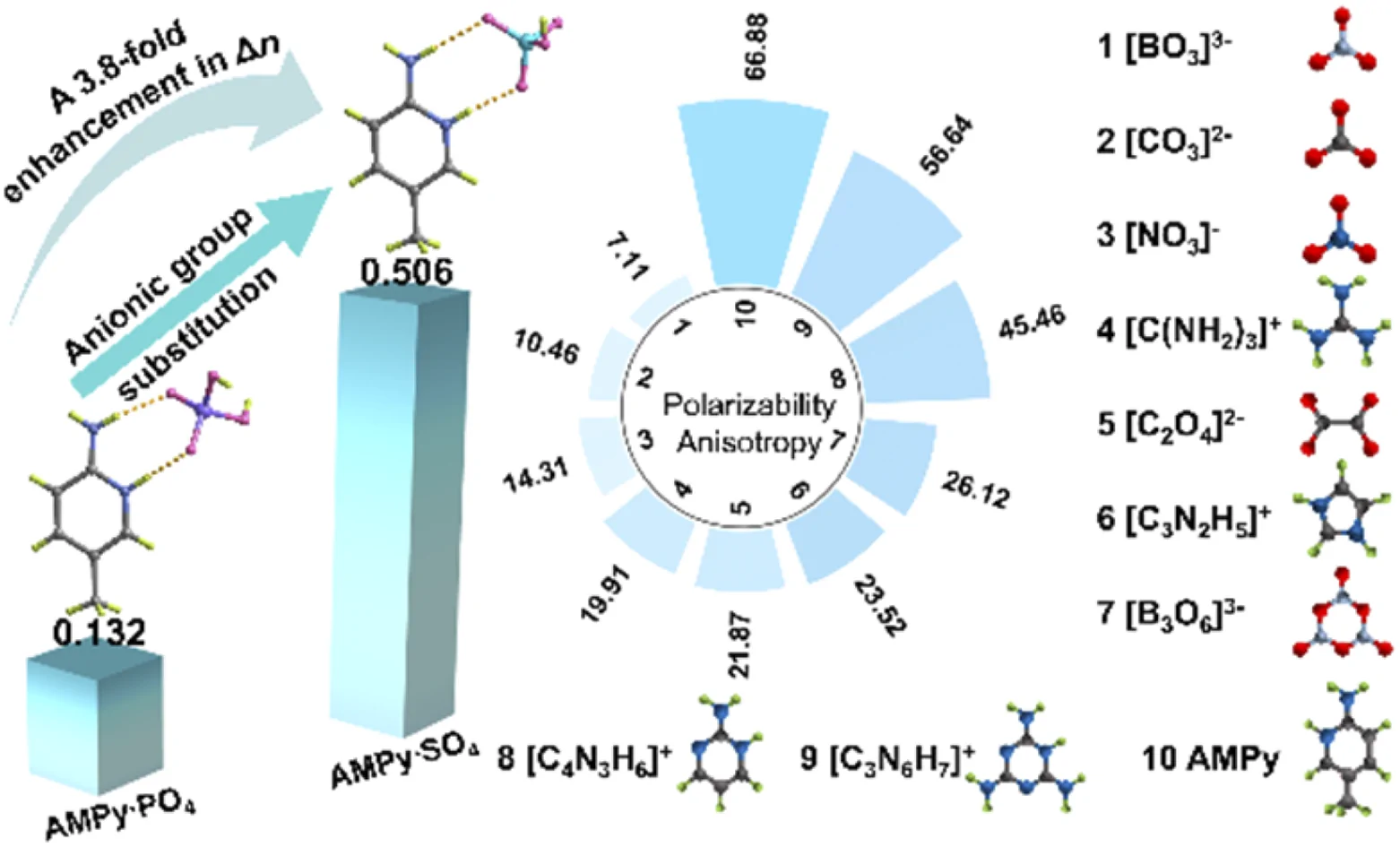

## Introduction

Birefringent crystals inherently split incident light into two orthogonally polarized beams with different refractive indices, so they have been widely applied in advanced optical technologies such as polarimetry, laser modulation, optical communication and imaging systems.^1–7^ The extensively used commercial birefringent crystals include CaCO_3_, α-BaB_2_O_4_, YVO_4_, and TiO_2_, exhibiting a birefringence (Δ*n*) of 0.172@532 nm,^8^ 0.122@546 nm,^9^ 0.204@532 nm,^10^ and 0.256@546 nm,^11^ respectively. To meet the requirements for the miniaturization of optical devices and the development of high-resolution optical systems,^12,13^ high-birefringence crystals are urgently needed for developing high-performance polarization-based optical devices.^14,15^ Specifically, giant birefringence (Δ*n* > 0.3) in polarizing crystals is practically significant for reducing the size of polarization optical devices, since the same optical path difference can be achieved with thinner crystals. Nevertheless, conventionally available birefringent crystals exhibit birefringence lower than 0.3. Driven by rising industrial requirements, exploring novel birefringent crystals is of great scientific and technological significance.

It is well established that pronounced structural anisotropy favors substantial optical anisotropy and consequently yields high birefringence for crystals. From the perspective of structure–property relationships, structural anisotropy of a crystal is tightly correlated with the fundamental functional units and their spatial arrangement within the crystal packing.^16–20^ Incorporation of metal ions with stereochemically active lone pairs into crystal lattices constitutes an efficient route toward improved structural anisotropy.^21–24^ Typical species such as Sb^3+^, Sn^2+^, and Pb^2+^ possess uneven electron distribution, which induces asymmetric coordination geometries and structural distortions and thereby facilitates prominent structural anisotropy,^25–27^ as exemplified by (C_10_H_6_NO_2_)_2_SbF (0.87@546 nm),^28^ Sn_2_PO_4_I (0.664@ nm),^29^ [(H-cmpy)_4_(Pb_3_Br_10_)] (0.315@550 nm),^30^ and K_2_SbMoO_2_F_7_ (0.220@ nm).^31^ Although this represents a promising strategy for constructing high-birefringence crystals, it should be acknowledged that metal-containing crystals may induce biological and environmental toxicity and produce narrow band gaps unfavorable for short-wavelength applications.^32–34^ Accordingly, the fabrication of metal-free birefringent crystals with a high degree of birefringence has emerged as a new research trend. On the other hand, π-conjugated groups with delocalized π electrons possessing large polarization anisotropy are the typical birefringent active groups for construction of birefringent crystals.^35^ The inorganic π-conjugated groups of CO_3_^2−^, BO_3_^3−^, and NO_3_^−^ are the extensively explored groups.^36–39^ Recently, several organic π-conjugated species such as C_2_O_4_^2−^, [C(NH_2_)_3_]^+^, and pyridine derivatives have been identified as birefringence-active groups,^40–45^ which makes them highly promising candidates for building high-birefringence crystals.

As for the non-π-conjugated sulfate groups, they are regarded as unfavorable structural motifs for constructing birefringent crystals, because the high symmetry of their ideal tetrahedron gives rise to weak optical anisotropy. Consequently, the obtained sulfate crystals generally show low birefringence,^46,47^ rendering them unfavorable as birefringent candidates. One strategy to enhance the birefringence in sulfate crystals is to substitute oxygen with other atoms or groups,^48^ thereby fundamentally increasing the anisotropy of the modified sulfate groups. Substitution of an oxygen atom in the sulfate group by either a methyl or an amino group leads to an increase in the birefringence of the resultant crystals such as NH_4_SO_3_CH_3_ (0.03@546 nm) and (CN_4_H_7_)SO_3_NH_2_ (0.155@546 nm).^49^ Another approach to increasing birefringence in sulfate crystals is the incorporation of metal-centered polyhedra with significant structural distortion, which can generate crystals with large birefringence, such as CeF_2_(SO_4_) (0.361@546 nm)^50^ and [C(NH_2_)_3_]BiCl_2_SO_4_ (0.143@546 nm).^51^ Despite progress in sulfate-based birefringent crystals, it remains a major challenge to design sulfate crystals with sufficient birefringence.

**Fig. 1:**
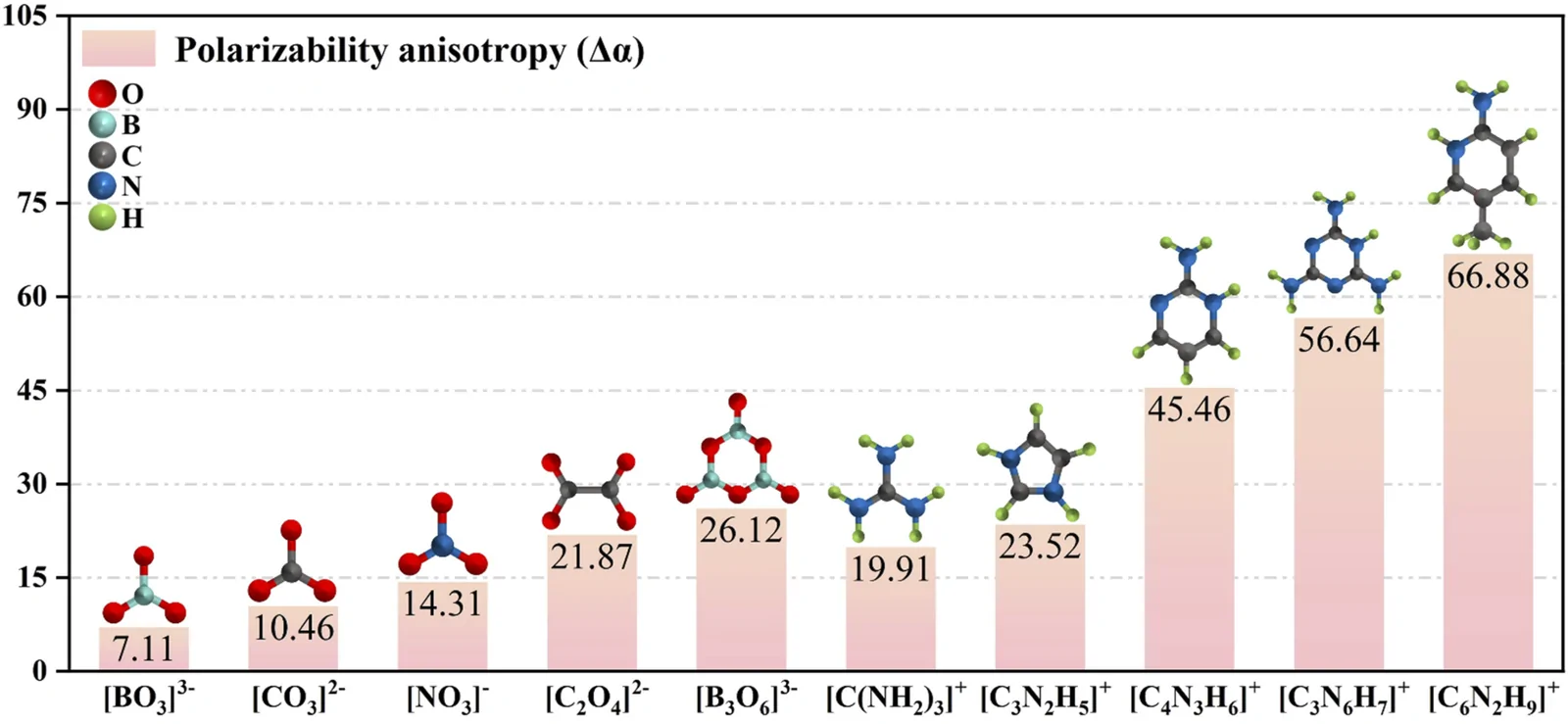
Comparison of the calculated polarizability anisotropy (Δ*α*) for typical π-conjugated groups.

In this work, we developed a strategy for designing metal-free birefringent crystals *via* integrating π-conjugated organic groups and non-π-conjugated tetrahedral inorganic groups. 2-Amino-5-methylpyridine (AMPy), serving as a birefringence-preferential unit, is selected by virtue of its prominent polarizability anisotropy (Δ*α*) (SI), superior to all conventional inorganic and organic π-conjugated groups (Fig. 1). Its large Δ*α* arises from extended π-conjugation and a strong, unimpeded D–π–A charge transfer along the molecular long axis, and the 5-methyl group enhances axial polarizability. This combination ensures a pronounced axial-to-transverse polarizability difference, making AMPy a superior building block for birefringent materials. However, π-conjugated groups can irreversibly narrow the band gap of the resultant crystals due to the conjugation effects.^4,52^ Given that non-π-conjugated tetrahedral groups (*e.g.*, phosphate and sulfate) contribute to a wide bandgap,^53,54^ crystals with broad optical transparency and large birefringence can be created by assembling AMPy with tetrahedral acidic groups. Besides, because pyridine nitrogen atoms are frequently protonated, acidic groups serve as anionic sources for achieving charge balance. Here, two crystals, (C_6_H_9_N_2_)(H_2_PO_4_) and (C_6_H_9_N_2_)(HSO_4_) (termed AMPy·PO_4_ and AMPy·SO_4_, respectively) are presented. For the first time, 2-amino-5-methylpyridine is identified as a novel birefringent active group, while the synergy of the organic and inorganic constituents achieves superior optical properties. Although non-π-conjugated tetrahedral groups exhibit weak optical anisotropy, they link π-conjugated birefringence-active moieties that govern the optical anisotropy through directional hydrogen bonds, thereby dominating the spatial arrangement of structural units and ultimately influencing the optical properties of the obtained crystals. Notably, upon substitution of the tetrahedral group from phosphate to sulfate, a 3.8-fold enhancement in birefringence is observed, boosting giant birefringence of 0.506 at 550 nm for the sulfate crystal. This value rivals those of all state-of-the-art sulfate crystals and highlights the distinct roles of the different tetrahedral groups.

## Results and discussion

**Fig. 2:**
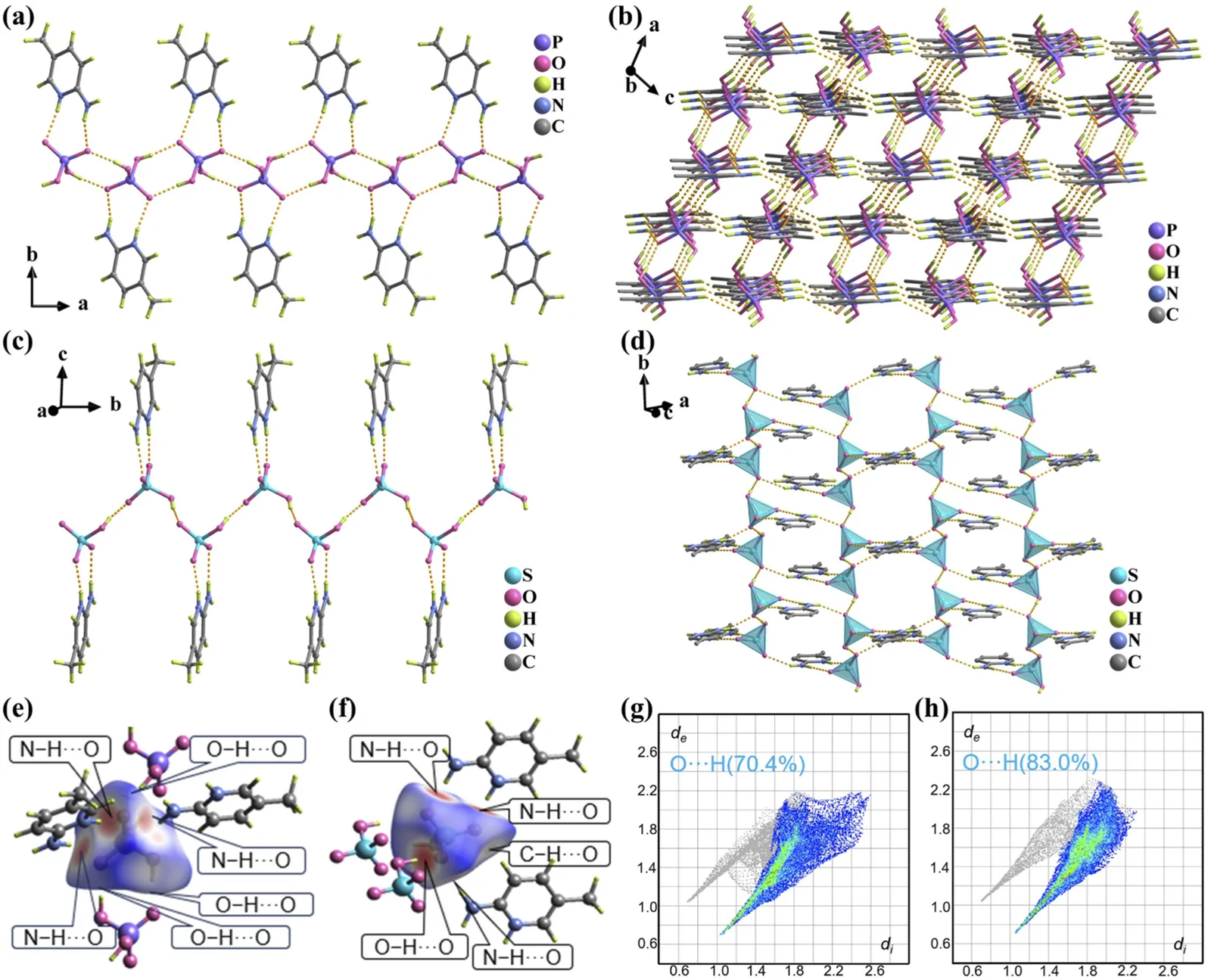
(a) 1-D and (b) 3-D structures of AMPy·PO_4_. (c) 1-D and (d) 2-D structures of AMPy·SO_4_. Hirshfeld *d*_norm_ surfaces of (e) H_2_PO_4_^−^ in AMPy·PO_4_ and (f) HSO_4_^−^ in AMPy·SO_4_ (red regions represent O–H⋯O and N–H⋯O bonds). 2-D fingerprint plots for H⋯O contacts in (g) AMPy·PO_4_ and (h) AMPy·SO_4_.

AMPy·PO_4_ and AMPy·SO_4_ were obtained by slow evaporation of aqueous mixtures of 2-amino-5-methylpyridine with phosphoric acid and sulfuric acid (SI), respectively. AMPy·PO_4_ is crystallized in a monoclinic space group *P*2_1_/*n* (Table S1 in the SI). A H_2_PO_4_^−^ anion and a (C_6_N_2_H_9_)^+^ cation are present in the asymmetric unit of AMPy·PO_4_. Each H_2_PO_4_^−^ unit is hydrogen-bonded with two H_2_PO_4_^−^ units through four hydrogen bonds (Table S2) to generate a one-dimensional (1-D) chain-like structure extending along the *a* axis (Fig. 2a). The (C_6_N_2_H_9_)^+^ cations are pendently attached to the 1-D chain *via* N–H⋯O hydrogen bonds (Fig. 2a and Table S2), which are arranged on both sides of the 1-D chain. The 1-D chain is connected to four adjacent chains by N–H⋯O hydrogen bonds (Fig. S1a and Table S2) to give a three-dimensional (3-D) hydrogen-bonding (HB) structure (Fig. 2b). In the 3-D structure, the adjacent chains are interdigitated with each other, which enables the (C_6_N_2_H_9_)^+^ protrusions of the 1-D chain interdigitate with adjacent chains, forming π⋯π interactions between the offset parallel pyridine rings from adjacent chains (Fig. S1b). Thus, the 3-D architecture is further complemented by noncovalent spatial interlocking. The synergistic combination of hydrogen bonds with π⋯π interactions between the H_2_PO_4_^−^ and (C_6_N_2_H_9_)^+^ moieties reinforces the structural robustness of the 3-D framework.

Although AMPy·SO_4_ also crystallizes in the *P*2_1_/*n* space group, it has different unit-cell parameters from those of AMPy·PO_4_ (Table S1). The asymmetric unit of AMPy·PO_4_ contains one HSO_4_^−^ and one (C_6_N_2_H_9_)^+^. A HSO_4_^−^ anion is hydrogen bonded with two adjacent HSO_4_^−^ anions *via* two hydrogen bonds (Table S2) to form a 1-D sinusoidal chain propagating along the *b* axis (Fig. 2c), which is different from the straight phosphate chains in AMPy·PO_4_. Similarly, the (C_6_N_2_H_9_)^+^ moieties acting as the pendants are attached to the 1-D chain *via* N–H⋯O hydrogen bonds (Fig. 2c and Table S2), which are arranged on both sides of the 1-D chain. Each 1-D chain is connected to two adjacent chains through N–H⋯O hydrogen bonds (Fig. S2a and Table S2) to generate a 2-D layered structure extending along the (1 0 −1) plane (Fig. 2d). The (C_6_N_2_H_9_)^+^ units are located on both sides of the 2-D layer (Fig. S2c). All pyridine planes located on the same side of the layer are parallel to each other, while the pyridine planes on the different sides of a layer are almost parallel to each other with a small dihedral angle of 8° (Fig. S2c).

To better understand the importance of the hydrogen bonds in the construction of the structures, Hirshfeld surface analysis derived from CrystalExplorer software was used for elucidating the HB interactions.^55^ The Hirshfeld *d*_norm_ surfaces plots for the anionic groups in AMPy·PO_4_ and AMPy·SO_4_ show that the HB interactions hold the anionic and cationic constituents together (Fig. 2e and f). More importantly, the corresponding 2-D fingerprint plots show that H⋯O hydrogen bonds are dominated in the surface areas, which are 70.4 and 83.0% of the total surface areas for AMPy·PO_4_ and AMPy·SO_4_ (Fig. 2g and h), respectively. Such results imply that the HB interactions are the primary interactions between the anionic and cationic constituents in both compounds, emphasizing the pivotal roles of the hydrogen bonds in stabilizing the structures.

The purity of the as-synthesized crystals was examined by powder X-ray diffraction (XRD). The experimental powder XRD patterns were in good agreement with the simulated ones derived from the single-crystal structures, confirming that both compounds are phase-pure (Fig. S3). AMPy·PO_4_ and AMPy·SO_4_ are thermally stable up to 203 and 195 °C (Fig. S4), respectively. The UV-vis-NIR diffuse reflectance spectra of AMPy·PO_4_ and AMPy·SO_4_ were measured, which display UV cut-off edges at 322 nm and 357 nm, giving rise to the experimental band gaps of 3.37 eV and 3.20 eV, respectively (Fig. S5). The wide band gaps observed for both compounds substantiate that non-π-conjugated groups effectively preserve a large bandgap upon incorporation into π-conjugated systems, thereby validating our design strategy.

**Fig. 3:**
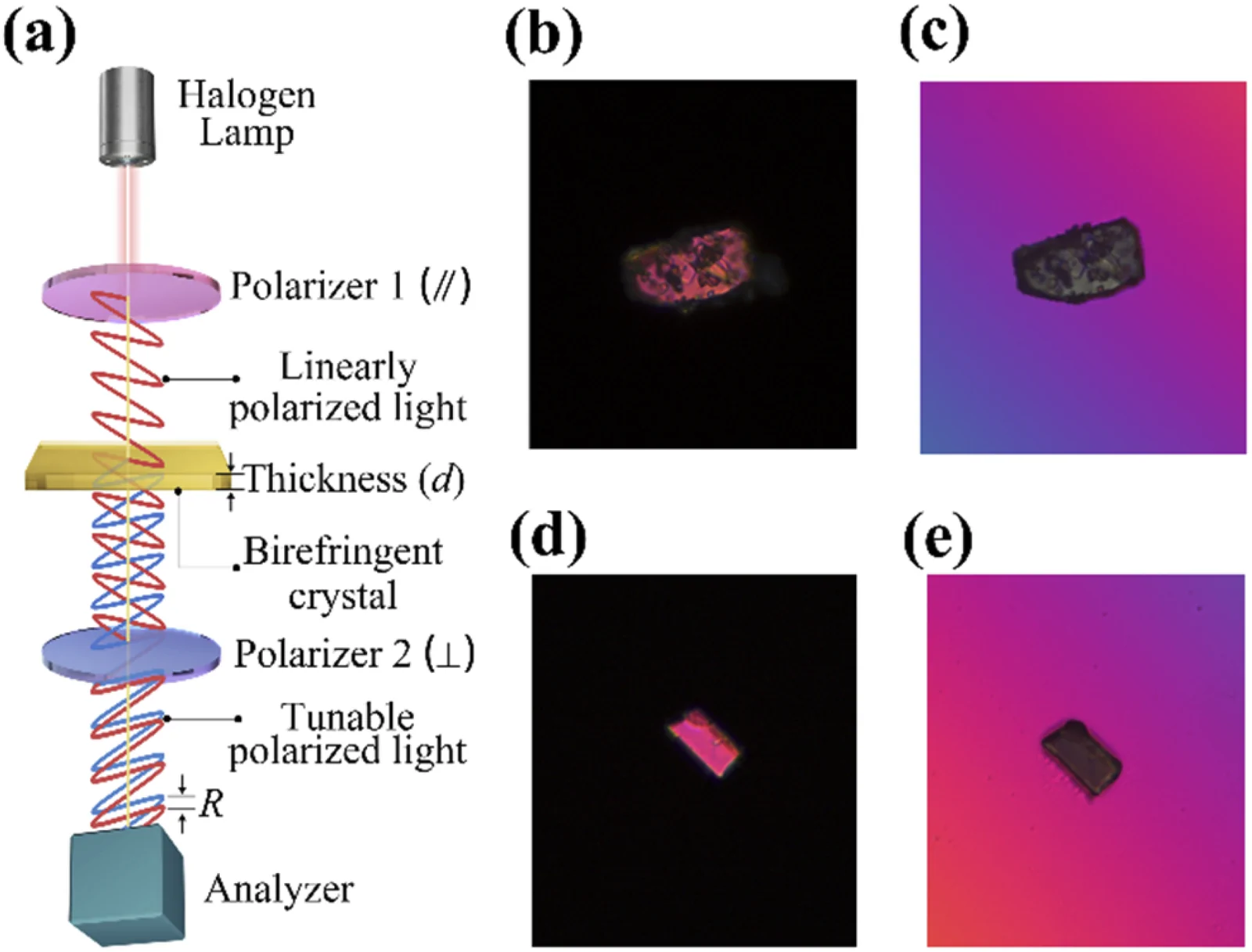
(a) Schematic showing the optical path for measuring birefringence. Original interference colours for (b) AMPy·PO_4_ and (d) AMPy·SO_4_. Crystals of (c) AMPy·PO_4_ and (e) AMPy·SO_4_ under complete extinction.

**Fig. 4:**
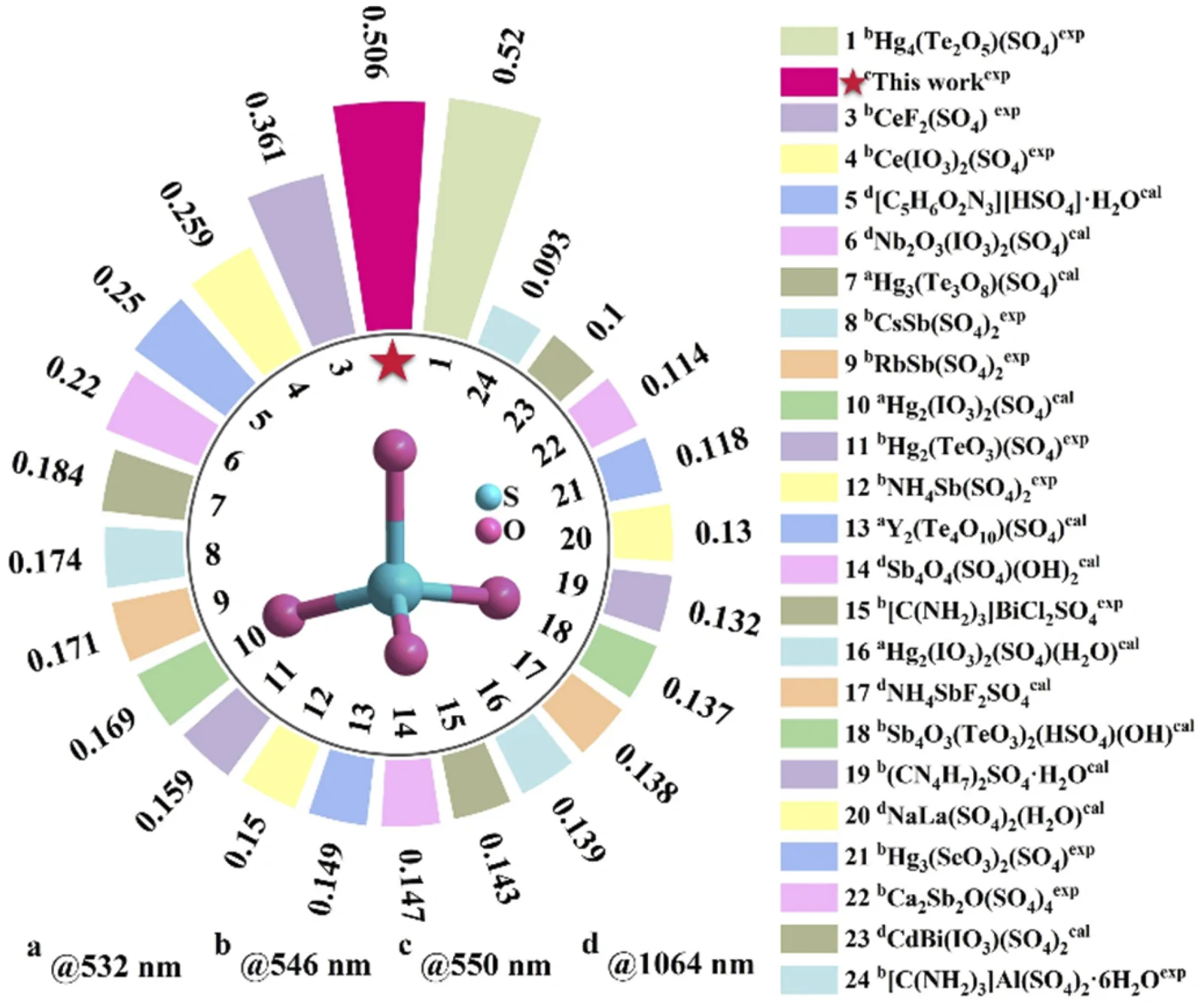
Comparison of the birefringence of the sulfate crystals (cal: calculated; exp: experimental) (birefringence at (a) 532 nm, (b) 546 nm, (c) 550 nm, and (d) 1064 nm).

Since *R* = *d* × Δ*n* (*R* = optical path difference, *d* = crystal thickness), thinner crystals with giant birefringence are beneficial for miniaturizing polarization optical devices. For example, a birefringent crystal inserted between two crossed polarizers induces an optical path difference, converting linearly polarized light into elliptically polarized light (Fig. 3a). To obtain a quarter-wave phase retardation (*λ*/4 = 137 nm at 550 nm), α-BaB_2_O_4_ (0.122@546 nm) requires a crystal thickness of about 1120 µm, whereas a crystal with Δ*n* = 0.5 needs only about 130 µm. This corresponds to an approximate order-of-magnitude reduction in the thickness of the polarization device. Using a polarization microscope equipped with a Berek compensator,^56,57^ the birefringence of the crystals was measured at a wavelength of 550 nm. The AMPy·PO_4_ crystal exhibits a second-order purple-red original interference color under crossed polarized light (Fig. 3b). Complete extinction for the crystal was achieved by rotating the quartz compensator (Fig. 3c). The *R* obtained from the compensator reading at extinction was 1.0733 µm, yielding a birefringence of 0.132 for AMPy·PO_4_ at 550 nm based on the thickness of 8.12 µm for the measured crystal (Fig. S6a). This birefringence value exceeds those of some commercial birefringent crystals, such as MgF_2_ (0.012@532 nm)^58^ and LiNbO_3_ (0.074@546 nm).^59^ In comparison with other phosphate birefringent crystals, the birefringence of AMPy·PO_4_ is moderate (Table S3 and Fig. S7). A second-order purple-red original interference color was observed for the AMPy·SO_4_ crystal under crossed polarized light (Fig. 3d). As the quartz compensator is rotated, the crystal of AMPy·SO_4_ reaches complete extinction (Fig. 3e). The *R* and *d* of the testing crystal were measured to be 1.0520 µm and 2.08 µm (Fig. S6b), respectively, resulting in a birefringence of 0.506 for the AMPy·SO_4_ crystal at 550 nm. Such remarkable birefringence of AMPy·SO_4_ surpasses that of all commercially available birefringent crystals, such as YVO_4_ (0.204@532 nm)^10^ and TiO_2_ (0.306@546.1 nm).^11^ Among the birefringent sulfate crystals, AMPy·SO_4_ ranks among the top in terms of birefringence (Fig. 4) and is second only to Hg_4_(Te_2_O_5_)(SO_4_) (0.520@546 nm), which contains highly distorted metal-centered polyhedra,^60^ demonstrating superior birefringent performance. Consequently, AMPy·SO_4_ is established herein as a new benchmark material among the metal-free birefringent sulfate crystals, placing it among the top-tier candidates for high-performance birefringent crystals (Table S4). Such findings indicate that the tetrahedral groups have the capability to regulate the spatial distribution of the functional π-conjugated groups through supramolecular interactions such as hydrogen bond and electrostatic interaction, establishing a feasible strategy to design high-performance birefringent semiorganic crystals.

**Fig. 5:**
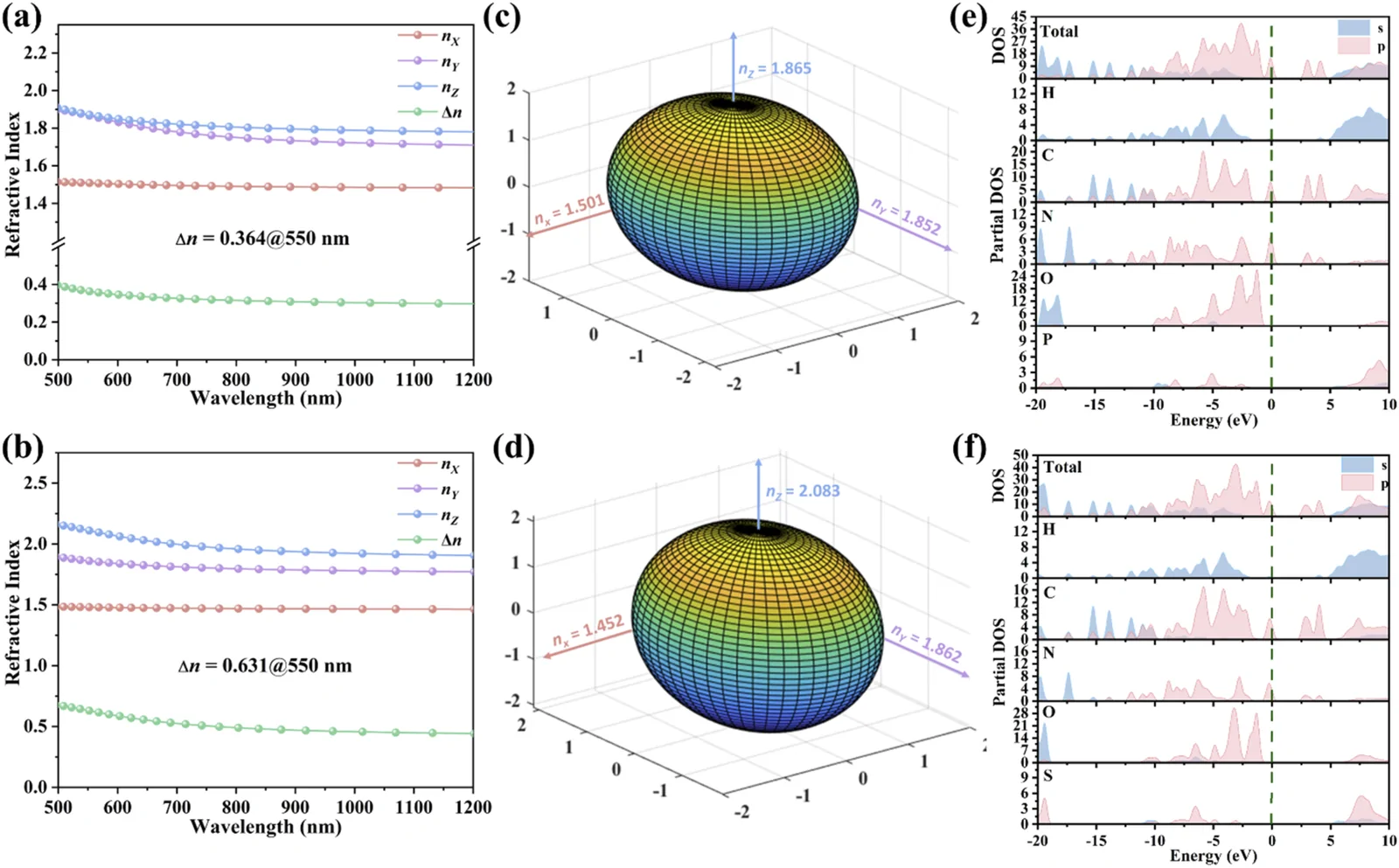
The refractive index curves for (a) AMPy·PO_4_ and (b) AMPy·SO_4_. Triaxial ellipsoids of three principal refractive indices at a wavelength of 550 nm for (c) AMPy·PO_4_ and (d) AMPy·SO_4_. Density of states for (e) AMPy·PO_4_ and (f) AMPy·SO_4_.

To verify the experimentally observed birefringence, we systematically investigated the refractive indices for both compounds through first-principles calculations based on Density Functional Theory (DFT). The calculated refractive indices of AMPy·PO_4_ at 550 nm are *n*_*X*_ = 1.501, *n*_*Y*_ = 1.852, and *n*_*Z*_ = 1.865 (Fig. 5a). Based on the nature of the biaxial crystal for both crystals, the Δ*n* for AMPy·PO_4_ can be calculated with Δ*n* = *n*_*Z*_ − *n*_*X*_, giving a Δ*n* of 0.364@550 nm for AMPy·PO_4_ (Fig. 5a). For AMPy·SO_4_, the Δ*n* is 0.631 at 550 nm based on *n*_*X*_ = 1.452, *n*_*Y*_ = 1.862, and *n*_*Z*_ = 2.083 (Fig. 5b). The calculated birefringence values for both crystals are in reasonable agreement with the experimental measurements. Fig. 5c and d show the refractive index ellipsoids corresponding to the three principal refractive indices of AMPy·PO_4_ and AMPy·SO_4_ at 550 nm, respectively. Compared with AMPy·PO_4_, the refractive index ellipsoid of AMPy·SO_4_ is significantly anisotropic, indicating that its structural anisotropy is markedly larger than that of AMPy·PO_4_, corroborating the larger birefringence of AMPy·SO_4_.

To reveal the intrinsic correlation between crystal structure and optical properties, structural analyses and band structure calculations were performed. Since layered structures produce the maximum difference between parallel and perpendicular layers, the layered arrangement of birefringence-active groups induces strong structural anisotropy, which effectively enhances birefringence.^35,61^ In AMPy·SO_4_, the HSO_4_^−^ group, acting as three hydrogen-bond acceptors and one hydrogen-bond donor, forms four HB interactions with two adjacent HSO_4_^−^ groups and one (C_6_N_2_H_9_)^+^ group (Fig. 2c). Directed by these HB interactions, AMPy·SO_4_ adopts a 2-D HB structure with π-conjugated (C_6_N_2_H_9_)^+^ units arranged nearly in parallel. By contrast, the H_2_PO_4_^−^ group serves as four hydrogen-bond acceptors and two hydrogen-bond donors, engaging in six HB interactions with two neighboring H_2_PO_4_^−^ groups and a (C_6_N_2_H_9_)^+^ cation (Fig. 2a). Governed by these interactions, the 3-D architecture of AMPy·PO_4_ is constructed. As a result, AMPy·SO_4_ possessing a 2-D HB layered arrangement displays more prominent structural anisotropy and consequently achieves enhanced birefringence. Furthermore, the two compounds have an equal count of π-conjugated (C_6_N_2_H_9_)^+^ units per unit cell. Given the larger unit cell volume of AMPy·PO_4_, AMPy·SO_4_ features denser stacking of the π-conjugated moieties. As a result, the densely packed birefringence-active groups within the layered structure give rise to the exceptional birefringence of AMPy·SO_4_.

The calculated results show that the theoretical band gaps of AMPy·PO_4_ and AMPy·SO_4_ are 2.91 eV and 2.63 eV, respectively (Fig. S8), both of which are lower than the experimental values of 3.37 eV and 3.20 eV. This deviation arises from the discontinuity of the exchange-correlation energy functional under the generalized gradient approximation (GGA), which leads to an underestimation of the band gap.^62^ The optical properties of materials are closely related to the electronic transition behavior near the band gap. In order to elucidate the microscopic origin of their optical performance, we systematically analyzed the electronic structures around the band gaps for the two compounds. Fig. 5e and f present the total and partial density of states (DOS and PDOS) of AMPy·PO_4_ and AMPy·SO_4_. It can be seen that the valence band maxima of both crystals are primarily contributed by the C 2p and N 2p orbitals of the organic ligands together with the O 2p orbitals of the inorganic anions. In contrast, the conduction band minima originate mainly from the C 2p and N 2p orbitals of the organic ligands, with negligible contribution from the inorganic anions. This indicates that the band gaps of both compounds are jointly determined by the π-conjugated (C_6_N_2_H_9_)^+^ moiety and the inorganic tetrahedral groups, which together dominate the optical properties of the crystals. In both compounds, the C 2p and N 2p orbitals show significant overlap in the same energy range, suggesting strong delocalized π-conjugation interactions within the organic cations.

**Fig. 6:**
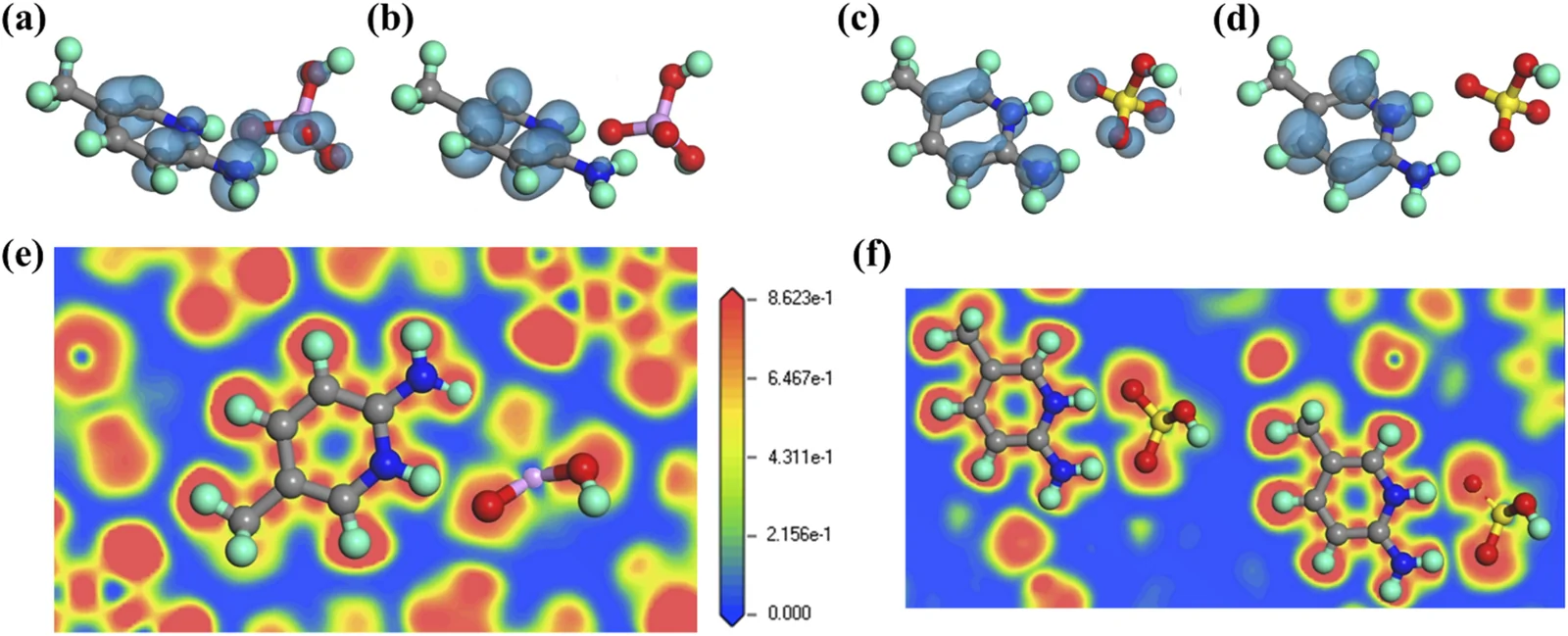
The HOMOs for (a) AMPy·PO_4_ and (c) AMPy·SO_4_. The LUMOs for (b) AMPy·PO_4_ and (d) AMPy·SO_4_. 2-D ELF maps of (e) AMPy·PO_4_ and (f) AMPy·SO_4_.

We further analyzed the distributions of the highest occupied molecular orbitals (HOMOs) and the lowest unoccupied molecular orbitals (LUMOs) of AMPy·PO_4_ and AMPy·SO_4_. For both compounds, the HOMOs are primarily composed of the C 2p and N 2p orbitals of the pyridine ring in the (C_6_N_2_H_9_)^+^ cation together with the O 2p orbitals of the inorganic anions (Fig. 6a and c). In contrast, the LUMOs are dominated by the hybridized C 2p and N 2p orbitals within the organic cation (C_6_N_2_H_9_)^+^ (Fig. 6b and d). These results indicate that both (C_6_N_2_H_9_)^+^ cations and the (H_2_PO_4_)^−^/(HSO_4_)^−^ anions make important contributions to the optical properties of the systems. Since the electron localization function (ELF) maps of AMPy·PO_4_ and AMPy·SO_4_ exhibit similar characteristics (Fig. 6e and f), AMPy·SO_4_ is taken as an example for analysis. In its ELF map parallel to the plane of the π-conjugated organic group, the ELF value gradually increases from the blue region to the red region, indicating that the system as a whole exhibits significant electron delocalization. Furthermore, the electron density around the oxygen atoms in (HSO_4_)^−^ displays a distinctly elliptical distribution. Such results further confirm that the large birefringence of both compounds originates from the planar π-conjugated organic cation (C_6_N_2_H_9_)^+^ and the synergistic electronic interaction between the organic cations and the tetrahedral inorganic anions.

## Conclusions

In conclusion, the organic π-conjugated AMPy and inorganic non-π-conjugated tetrahedral groups are synergistically integrated into single structures to yield two UV birefringent crystals, AMPy·PO_4_ and AMPy·SO_4_. In both compounds, the adjacent acidic groups are hydrogen-bonded with each other to give the 1-D chains. Under the guidance of the N–H⋯O hydrogen bonds, 1-D straight chains are linked by the protonated AMPy moieties to generate a 3-D structure for AMPy·PO_4_. By contrast, the zigzag chains in AMPy·SO_4_ are connected by AMPy cations to form a 2-D layer. Although the structural dimensionality of the two compounds is different, the parallel alignment of AMPy units is observed in both structures. Such favorable arrangement of the optically anisotropic AMPy groups in both structures results in the large birefringence for both compounds. In particular, AMPy·SO_4_ is identified as a new record structure for metal-free birefringent sulfate crystals and ranks highly among high-performance birefringent candidates. Structural analysis shows that the zigzag alignment of the sulfate groups optimizes the arrangement of the π-conjugated cationic AMPy groups guided by the N–H⋯O interactions, which reduces the dimensionality of the structure and increases the packing density of the AMPy groups, ultimately boosting the unprecedented birefringence of AMPy·SO_4_. Theoretical calculations demonstrate that optical anisotropy is primarily governed by the anisotropic AMPy moieties, and the synergistic coupling of inorganic components further dictates the optical properties. This work offers new insights into the exploration of metal-free semiorganic birefringent crystals and enables efficient modulation of the structural and optical characteristics of organic–inorganic birefringent crystals *via* inorganic group substitution.

## Author contributions

Yan-Zhen Xie: investigation, data curation, formal analysis, writing – original draft. Hai-Yan Jiang: methodology, writing – original draft. Yi Zhang: data curation, validation. Hui-Ping Xiao: data curation, validation. Qing-Yan Liu: methodology, supervision, funding acquisition, writing – review & editing. Yu-Ling Wang: conceptualization, supervision, funding acquisition, writing – review & editing.

## Conflicts of interest

There are no conflicts to declare.

## Supplementary Material

SC-OLF-D6SC05559A-s001

SC-OLF-D6SC05559A-s002

## Data Availability

CCDC 2565839 and 2565840 (AMPy·PO_4_ and AMPy·SO_4_) contain the supplementary crystallographic data for this paper.^63*a*,*b*^ The data supporting this article have been included as part of the supplementary information (SI). Supplementary information: the syntheses of compounds, crystal structure refinement data, details of theoretical calculations, additional crystal structures, thermogravimetric curves, UV-vis-NIR diffuse reflectance spectra, figures of the thickness of the testing crystals. See DOI: https://doi.org/10.1039/d6sc05559a.
