# Supplementary material for: Achieving record birefringence in metal-free semiorganic sulfate crystals via tetrahedral anionic group substitution
Source: Chem Sci. 2026 Aug 17;17(39):18924–32. doi: 10.1039/d6sc05559a (PMC13501470; doi:10.1039/d6sc05559a)
Supplement: Supplementary file 1 [file SC-017-D6SC05559A-s001.pdf]

Supplementary Information (SI) for Chemical Science.
This journal is © The Royal Society of Chemistry 2026

Electronic Supplementary Information (ESI)

Achieving record birefringence in metal-free semiorganic sulfate crystals via tetrahedra anionic group substitution

Yan-Zhen Xie,[a,‡] Hai-Yan Jiang,[a,‡] Yi Zhang,[a] Hui-Ping Xiao,[a] Qing-Yan Liu[*a,b] and Yu-Ling Wang[*a]

[a] Key Laboratory of Porous Functional Materials of Jiangxi Province, Key Lab of Fluorine and Silicon for Energy Materials and Chemistry of Ministry of Education, College of Chemistry and Materials, Jiangxi Normal University, Nanchang 330022, Jiangxi, P. R. China.
[b] Department of Ecological and Resources Engineering, Fujian Key Laboratory of Eco-industrial Green Technology, Wuyi University, Wuyishan 354300, Fujian, P. R. China.

[‡] These authors contributed equally to this work.
Corresponding Authors (email: qyliuchem@jxnu.edu.cn; ylwang@jxnu.edu.cn)

1. Experimental Section

Chemicals. All reagents were used as received without purification. 2-Amino-5-methylpyridine (98%) was purchased from Shanghai Bide Biotech Co., Ltd., China. Phosphoric acid (> 85 w% in H_2O) was purchased from Tianjin ZhiYuan Reagent Co., Ltd., China. Sulfuric acid (95-98 w% in H_2O) was purchased from Chengdu Kelong Chemical Co.,Ltd., China.

Synthesis of AMPy·PO_4. A slow evaporation solution method was employed for the synthesis of AMPy ·PO_4. 2-Amino-5-methylpyridine (0.043 g, 0.4 mmol) and phosphoric acid (> 85 w% in H_2O) (0.046 g, 0.4 mmol) were dissolved in 3 mL of water in a 25 mL beaker and sonicated for 10 minutes. The beaker was then sealed with plastic wrap and left to stand at room temperature. After two weeks of slow solvent evaporation, colorless crystals were obtained in approximately 83% yield. Elemental analysis for AMPy·PO_4 (206.14) (calc/found: H, 5.38/5.42; C, 34.96/34.91; N,

13.59/13.52). IR data (KBr, cm^{-1}): 3392 (m), 2387 (w), 1678 (s), 1481 (m), 1254 (s), 1107 (s), 955 (s), 821 (s), 553 (s).

Synthesis of AMPy·SO_4. AMPy·SO_4 was synthesized following a procedure similar to that for AMPy·PO_4, but with phosphoric acid replaced by sulfuric acid (95-98 w% in H_2O) (0.041 g, 0.4 mmol). Colorless transparent crystals were obtained at room temperature in 85% yield. Elemental analysis for AMPy·SO_4 (206.22) (calc/found: H, 4.89/4.87; C, 34.94/34.91; N, 13.58/13.48). IR data (KBr, cm^{-1}): 3335 (m), 3154 (m), 2764 (w), 1664 (s), 1620 (s), 1475 (m), 1350 (s), 1020 (m), 825 (s), 560 (s).

2. Characterization.

Physical Measurements. FT-IR spectra were recorded from KBr disc on a Perkin-Elmer Spectrum One FT-IR spectrometer ranging from 400 to 4000 cm^{-1}. Thermogravimetric analyses were performed under a nitrogen atmosphere with a heating rate of 10 °C/min using a PE Diamond thermogravimetric analyser. Powder X-ray diffraction analyses were performed on a Rigaku DMAX 2500 diffractometer with Cu-Kα radiation (λ = 1.5418 Å). The UV-vis-NIR diffuse reflection data was recorded at room temperature using $BaSO_4$ powder sample as a standard (100% reflectance) on a PerkinElmer Lamda-950 UV/vis/NIR spectrophotometer (scanning wavelength is between 200 and 800 nm).

Crystallographic Study. X-ray single-crystal diffraction experiment was carried out a Rigaku Oxford SuperNova diffractometer equipped with an EOS detector (Mo-Kα radiation, λ = 0.71073 Å). Absorption correction and data reduction were handled with a *CrysAlisPro package*.[1] The *SHELXT-2015*[2] and *SHELXL-2018*[3] were applied to structure solution and refinement. The hydrogen atoms were added theoretically. Non-hydrogen atoms were refined anisotropically. Details of crystal parameters, data collection, and structure refinement are summarized in Table S1. The parameters of hydrogen bonds for both compounds are listed in Table S2.

Birefringence Measurement. The birefringence of crystal AMPy·PO_4 and AMPy·SO_4 was obtained through a polarizing microscope (Nikon LV1000) equipped with a Berek compensator at a wavelength of 550 nm. The following formula was listed to calculate birefringence: $R = |N_e\text{-}N_o| = \Delta n \times d$, where R denotes the optical path difference, Δn represents birefringence, and d denotes the thickness of the crystal.

Computational Methods. The first-principles calculations for the two compounds were performed by CASTEP [4] on a plane-wave pseudopotential total energy package based density functional theory (DFT).[5] The functional developed by Perdew-Burke-Ernzerhof (PBE) functional within the generalized gradient approximation (GGA) [6,7] form was adopted to describe the exchange-correlation energy. The ultrasoft pseudopotentials were used to model the effective interaction between atom cores and valence electrons. H $1s^1$, C $2s^22p^2$, N $2s^22p^3$, O $2s^22p^4$, P $3s^24p^3$ and S $3s^23p^4$ electrons were treated as valence electrons. The kinetic energy cutoff of 380 eV and dense 2 × 2 × 1 Monkhorst-Pack [8] k-point meshes in the Brillouin zones were chosen. The linear optical properties were examined based on the dielectric function $\varepsilon(\omega) = \varepsilon_1(\omega) + i\varepsilon_2(\omega)$. The imaginary part of dielectric function ε_2 can be calculated based on the electronic structures and the real part is obtained by the Kramers-Kronig transformation, accordingly the refractive indices and the birefringence (Δn) can be calculated. The frequency-dependent refractive indices were calculated to demonstrate the validity of birefringence measurements.

The polarizability anisotropy ($\Delta\alpha$) was acquired from static polarization (α) according to the following formula.[9] The static polarization is calculated using the DFT method based on the crystal data.

$$\Delta\alpha = \sqrt{[(\alpha_{xx}-\alpha_{yy})^2+(\alpha_{xx}-\alpha_{zz})^2+(\alpha_{yy}-\alpha_{zz})^2+6\left(\alpha_{xy}^2+\alpha_{xz}^2+\alpha_{yz}^2\right)]/2}$$

Table S1 Crystal data and structural refinement data

	AMPy·PO_4	AMPy·SO_4
Identificant on	$(C_6N_2H_9)(H_2PO_4)$	$(C_6N_2H_9)(HSO_4)$
Empirical formula	$C_6H_{11}N_2O_4P$	$C_6H_{10}N_2O_4S$
Formula weight	206.14	206.22
Temperature (*K*)	293(2)	298(2)
Crystal system	Monoclinic	Monoclinic
Space group	$P2_1/n$	$P2_1/n$
Z	4	4
a (Å)	7.9811(3)	11.5179(5)
b (Å)	10.9199(4)	6.8095(3)
c (Å)	11.1466(4)	12.0136(5)
α (°)	90	90
β (°)	108.456(4)	105.249(4)
γ (°)	90	90
V (Å^3)	921.49(6)	909.07(7)
Calculated density (g/cm^3)	1.486	1.507
Absorption coefficient (mm^{-1})	0.284	0.342
F(000)	432	432
Reflections collected	9210	9326
Goodness-of-fit on F^2	1.070	1.063
Final *R* indices	R_1 = 0.0486;	R_1 = 0.0524;
[$I > 2\sigma(I)$]^a	wR_2 = 0.1232	wR_2 = 0.1296
R indices	R_1 = 0.0579;	R_1 = 0.0700;
(all data)	wR_2 = 0.1291	wR_2 = 0.1417

^{a} $R_1 = \Sigma||F_o| - |F_c|| / \Sigma|F_o|$ and $wR_2 = \{\Sigma[w(F_o^2 - F_c^2)^2] / \Sigma[w(F_o^2)^2]\}^{1/2}$

Crystallographic data have been deposited at the Cambridge Crystallographic Data Centre (CCDC numbers are 2565839 and 2565840 for AMPy·PO_4 and AMPy·SO_4, respectively) and can be obtained from the Cambridge Crystallographic Data Centre.

Table S2 Hydrogen bond parameters of AMPy·PO_4 and AMPy·SO_4

AMPy·PO_4

D–H···A [Å]	d(D–H) [Å]	d(H···A) [Å]	d(D···A) [Å]	∠DHA[°]
O3–H3A···O1[#1]	0.82	1.73	2.545(2)	169.2
O4–H4···O2[#2]	0.82	1.82	2.611(2)	162.3
N1–H1···O2	0.86	1.95	2.805(2)	170.4
N2–H2A···O1	0.86	2.00	2.791(2)	153.3
N2–H2B···O2[#3]	0.86	2.32	3.069(2)	145.8

[a] Symmetry codes: #1: - *x* + 1, - *y*, - *z* + 1; #2: - *x*, - *y*, - *z* + 1; #3: *x* + 1/2, - *y* + 1/2, *z* + 1/2.

AMPy·SO_4

D–H···A [Å]	d(D–H) [Å]	d(H···A) [Å]	d(D···A) [Å]	∠DHA[°]
O4–H4···O3[#1]	0.82	1.78	2.592(3)	170.4
N1–H1···O1	0.86	1.90	2.751(3)	170.8
N2–H2A···O2	0.86	2.09	2.951(3)	179.2
N2–H2B···O3[#2]	0.86	2.17	2.983(3)	157.3

[a] Symmetry codes: #1: -*x* + 3/2, *y* - 1/2, - *z* + 3/2; #2: *x* - 1/2, - *y* +3 /2, *z* -1/2.

Table S3 Comparisons of the birefringent phosphate crystals.

Compounds	Birefringence	Ref.
$C_6N_7(NH_2)_3{\cdot}H_3PO_4$	0.609@1064 nm[a]	10
$(C_6H_6NO_2)H_2PO_4$	0.284@546 nm[b]	11
$(C_5H_6ON)(H_2PO_4)$	0.25@1064 nm[a]	12
$(C_6H_7N_2O)(H_2PO_4)$	0.191@1064 nm[a]	11
$(C_3H_5N_2)(H_2PO_4)$	0.15@546 nm[b]	13
$(C_6N_2H_9)(H_2PO_4)$	0.132@550 nm[b]	**This work**
$(C_4H_7N_2)(H_2PO_4)$	0.12@546 nm[b]	13
$[C(NH_2)_3]_6(PO_4)_2{\cdot}3H_2O$	0.078@546 nm[b]	14
$Pb_2(H_2PO_4)_2(C_2O_4)$	0.129@550 nm[b]	15
$Sr_2(H_2PO_4)_2(C_2O_4)$	0.107@550 nm[b]	15
$Na_3Sc_2(PO_4)_2F_3$	0.0978@546.1 nm[b]	16

$KTiOPO_4$	0.0927@1064 nm[b]	17
$Ba_3Sb_2(PO_4)_4$	0.086@532 nm[a]	18
$SrSn(PO_4)(H_2PO_4)$	0.08@1064 nm[a]	19
$BaSn_2(PO_4)_2$	0.07@1064 nm[a]	20
$K_2NaSbP_2O_8$	0.07@1024 nm[a]	21
$LiHgPO_4$	0.068@1064 nm[a]	22
β-$Cd(PO_3)_2$	0.059@1064 nm[a]	23
α-$NaSb_3P_2O_{10}$	0.053@1024 nm[a]	24
$Cd_3Sb_2(PO_4)_4(H_2O)_2$	0.053@532 nm[a]	18
$Sn^{II}Sn^{IV}(PO_4)_2$	0.048@1064 nm[a]	19
$La(PO_3)_3$	0.04@1064 nm[a]	23
KH_2PO_4	0.04@500 nm[a]	24
β-$Li_2RbBi(PO_4)_2$	0.0348@1064 nm[a]	24
$Rb_3PbBi(P_2O_7)_2$	0.031@1064 nm[a]	25
$KBe[PO_3(OH)]F$	0.025@1064 nm[a]	26
$Cs_3PbBi(P_2O_7)_2$	0.02@1064 nm[a]	25
$K_2SrP_4O_{12}$	0.016@1064 nm[a]	27
$K_4Mg_4(P_2O_7)_3$	0.0108@1064 nm[a]	28
$Rb_4Mg_4(P_2O_7)_3$	0.009@1064 nm[a]	28
$CsMgPO_4\cdot6H_2O$	0.005@1064 nm[a]	29
$RbMgPO_4\cdot6H_2O$	0.006@1064 nm[a]	29
$PbZn(PO_4)$	0.004@1064 nm[a]	30

a: Calculated value; b: experimental value.

Table S4 Comparisons of the birefringent sulfate crystals.

Compounds	Birefringence	Ref.
$Hg_4(Te_2O_5)(SO_4)$	0.520@546 nm[b]	31
$(C_6N_2H_9)$ (HSO_4)	0.506@550 nm[b]	**This work**
$CeF_2(SO_4)$	0.361@546 nm[b]	32
$Ce(IO_3)_2(SO_4)$	0.259@546 nm[b]	33
$[C_5H_6O_2N_3][HSO_4]\cdot H_2O$	0.25@1064nm[a]	34
$Nb_2O_3(IO_3)_2(SO_4)$	0.22@1064 nm[a]	35
$Hg_3(Te_3O_8)(SO_4)$	0.184@532 nm[a]	36
$CsSb(SO_4)_2$	0.174@546 nm[b]	37
$RbSb(SO_4)_2$	0.171@546 nm[b]	37
$Hg_2(IO_3)_2(SO_4)$	0.169@532 nm[a]	38
$Hg_2(TeO_3)(SO_4)$	0.159@546 nm[b]	31
$NH_4Sb(SO_4)_2$	0.150@546 nm[b]	39
$Y_2(Te_4O_{10})(SO_4)$	0.149@532 nm[a]	40
$Sb_4O_4(SO_4)(OH)_2$	0.147@1064 nm[a]	41
$[C(NH_2)_3]BiCl_2SO_4$	0.143@546 nm[b]	42
$Hg_2(IO_3)_2(SO_4)(H_2O)$	0.139@532 nm[a]	38
$NH_4SbF_2SO_4$	0.138@1064 nm[a]	43

$Sb_4O_3(TeO_3)_2(HSO_4)(OH)$	0.137@546 nm[a]	44
$Hg_2(SeO_3)(SO_4)$	0.133@532 nm[a]	44
$(CN_4H_7)_2SO_4 \cdot H_2O$	0.132@546.1 nm[a]	45
$Ga_2(TeO_3)(SO_4)(OH)_2$	0.122@532 nm[a]	36
$K_2SO_4 \cdot HIO_3$	0.14@589.3 nm[b]	46
$NaLa(SO_4)_2(H_2O)$	0.13@1064 nm[a]	47
$CsHgClSO_4 \cdot H_2O$	0.12@546 nm[a]	48
$Hg_3(SeO_3)_2(SO_4)$	0.118@546 nm[b]	49
$Ca_2Sb_2O(SO_4)_4$	0.114@546 nm[b]	39
$Rb_2SO_4 \cdot (SbF_3)_2$	0.11@1064 nm[a]	50
$RbSbSO_4Cl_2$	0.11@1064 nm[a]	51
$NH_3SO_3(NH_4)_2SO_4$	0.104@520 nm[a]	52
$CdBi(IO_3)(SO_4)_2$	0.1@1064 nm[a]	53
$Hg_3O_2SO_4$	0.1@546 nm[a]	48
$Zn_4(Te_3O_7)_2(SO_4)_2(H_2O)$	0.094@532 nm[a]	36
$[C(NH_2)_3]Al(SO_4)_2 \cdot 6H_2O$	0.093@546 nm[b]	54
$Y_3(TeO_3)_2(SO_4)_2(OH)(H_2O)$	0.092@532 nm[a]	40

a: Calculated value; b: experimental value.

3. Results and discussion

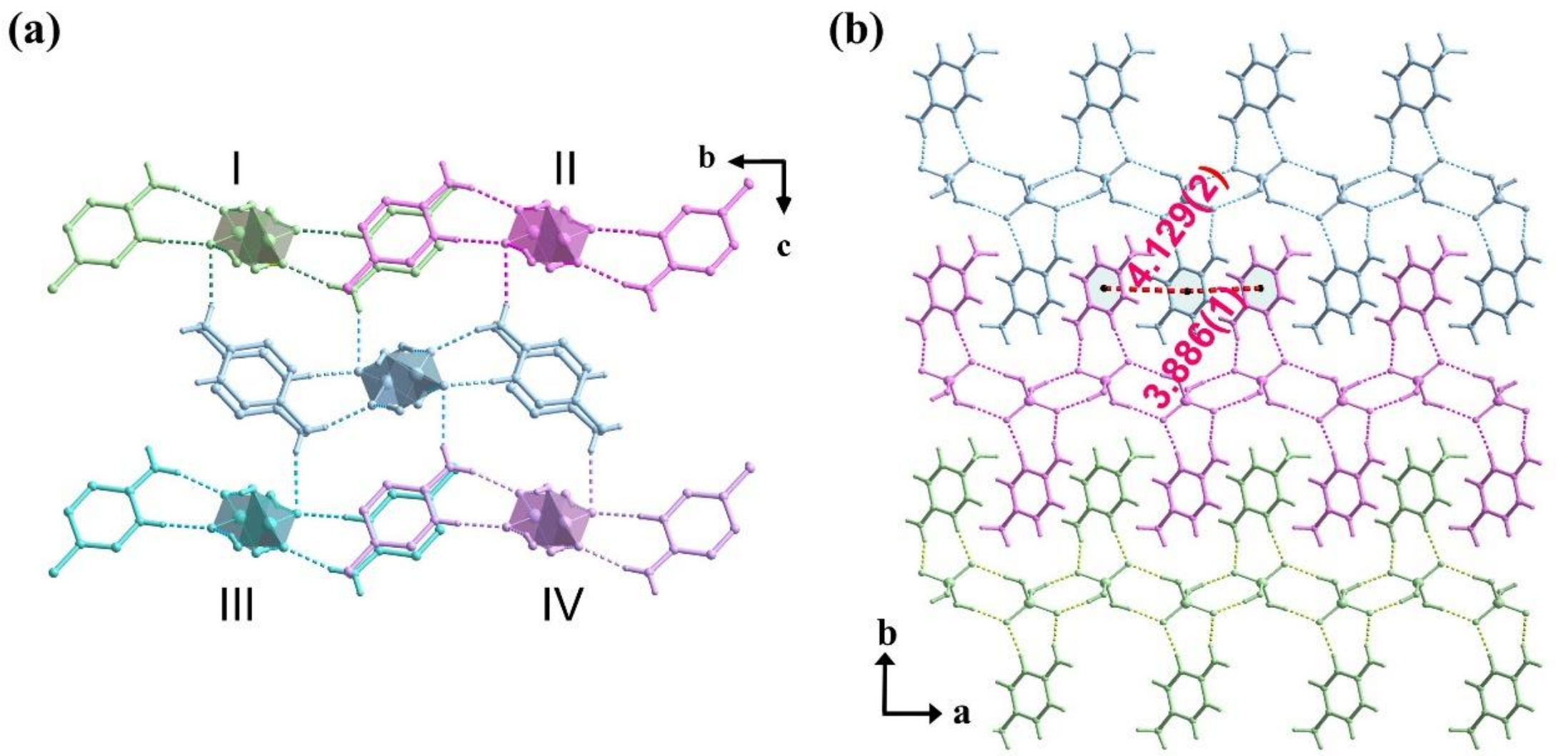

Fig. S1 (a) Each 1-D chain is connected to four adjacent chains (I, II, III, and IV) by N–H···O hydrogen bonds in AMPy ⱣO_4. (b) In the 3-D structure of AMPy ⱣO_4, the π···π interactions between the pyridine rings from adjacent chains are observed.

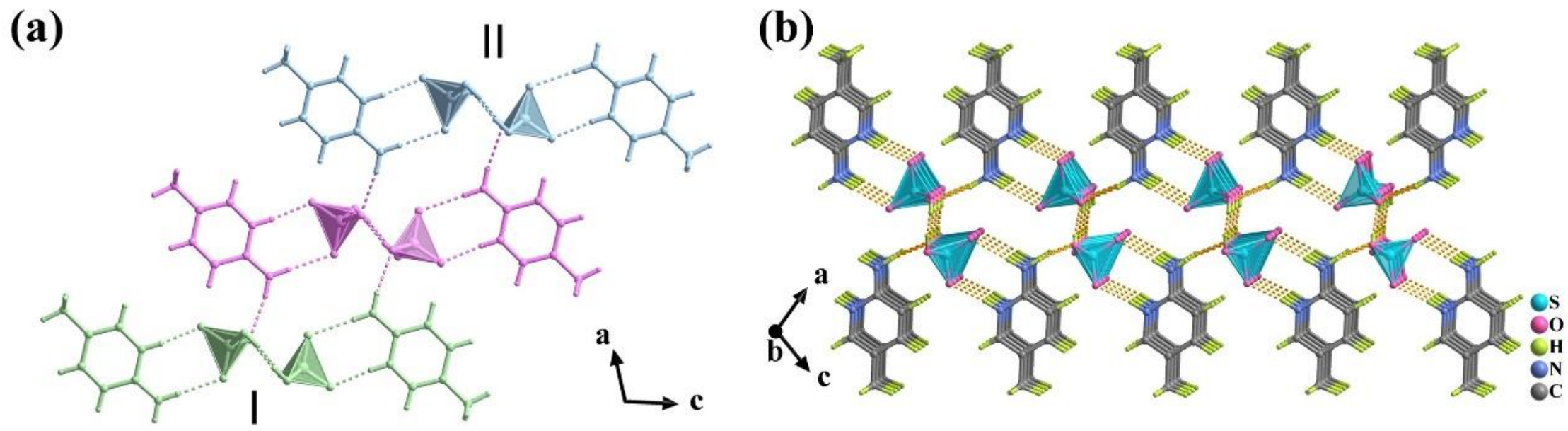

Fig. S2 (a) Each 1-D chain is connected to two adjacent chains (I and II) by N–H···O hydrogen bonds in AMPy·SO_4. (b) The 2-D layered structure for AMPy·SO_4.

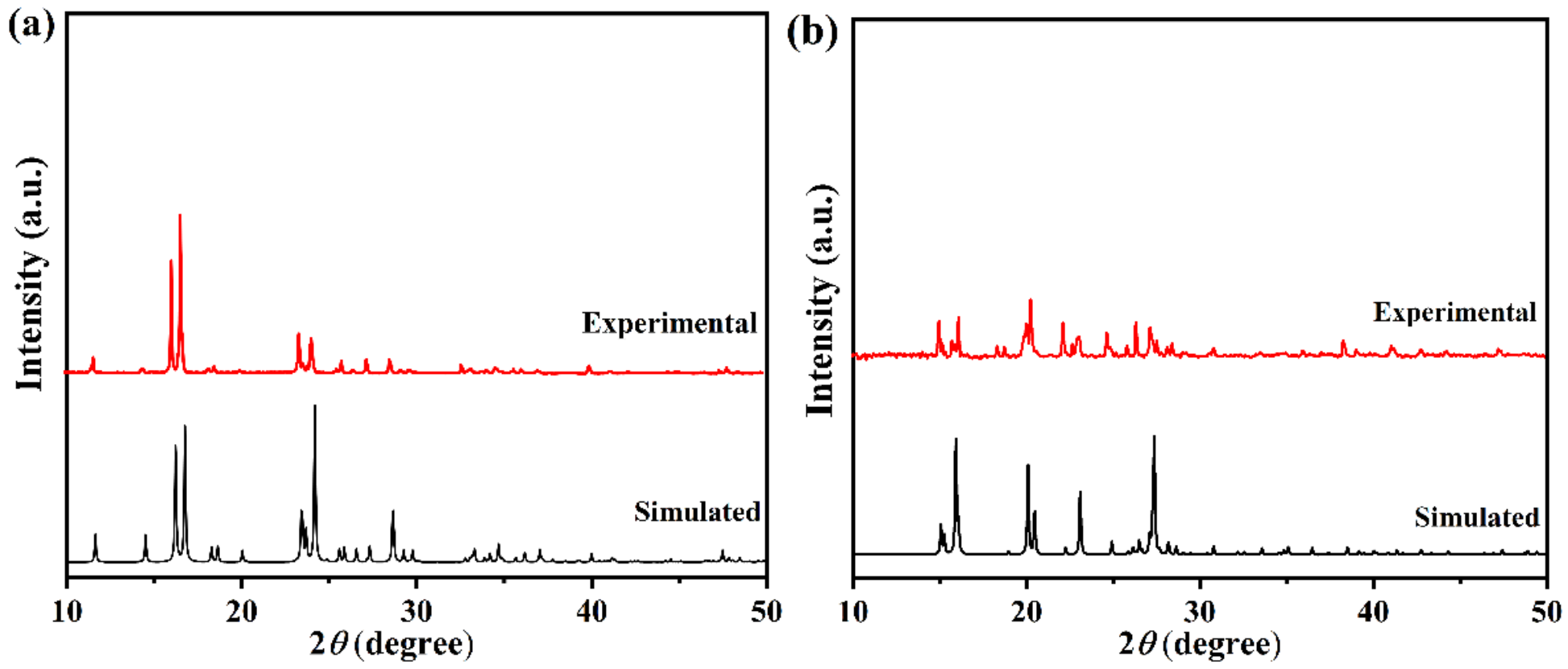

Fig. S3 PXRD patterns for compound (a) AMPy·PO_4 and (b) AMPy·SO_4.

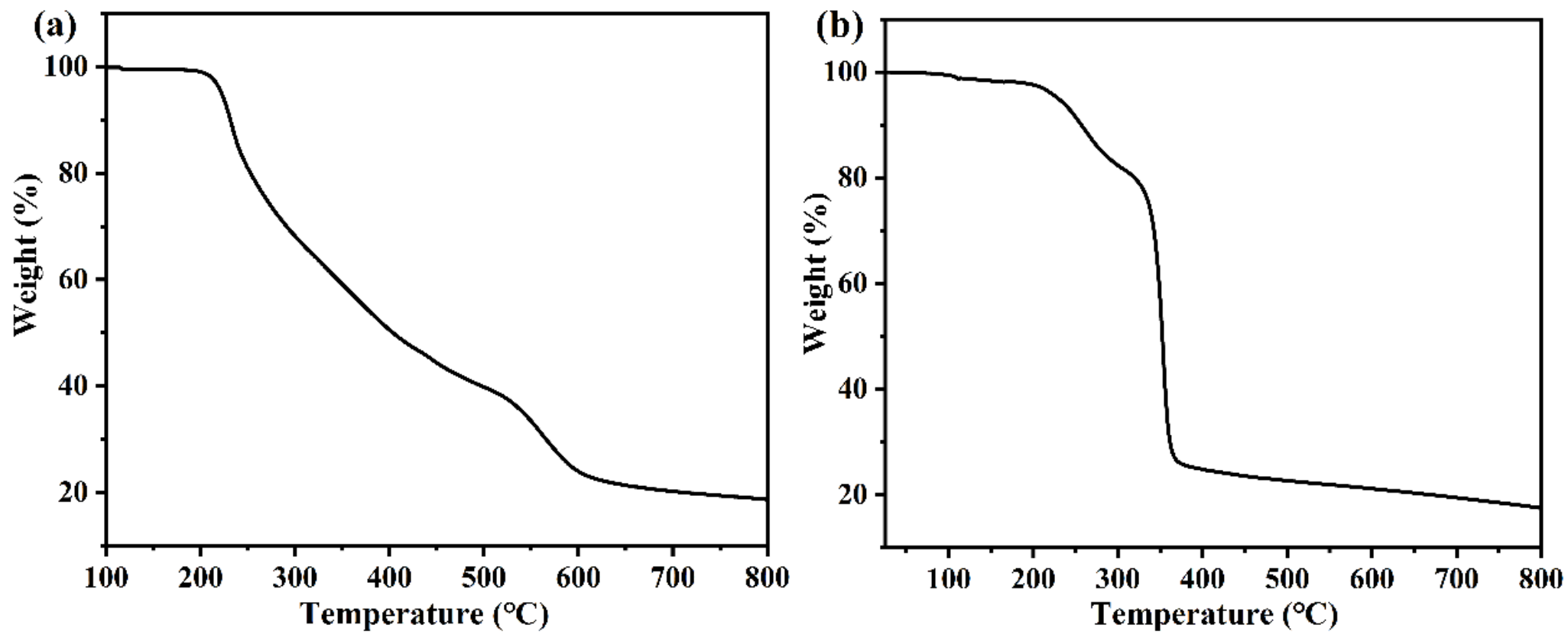

Fig. S4 TGA curves for (a) AMPy·PO_4 and (b) AMPy·SO_4.

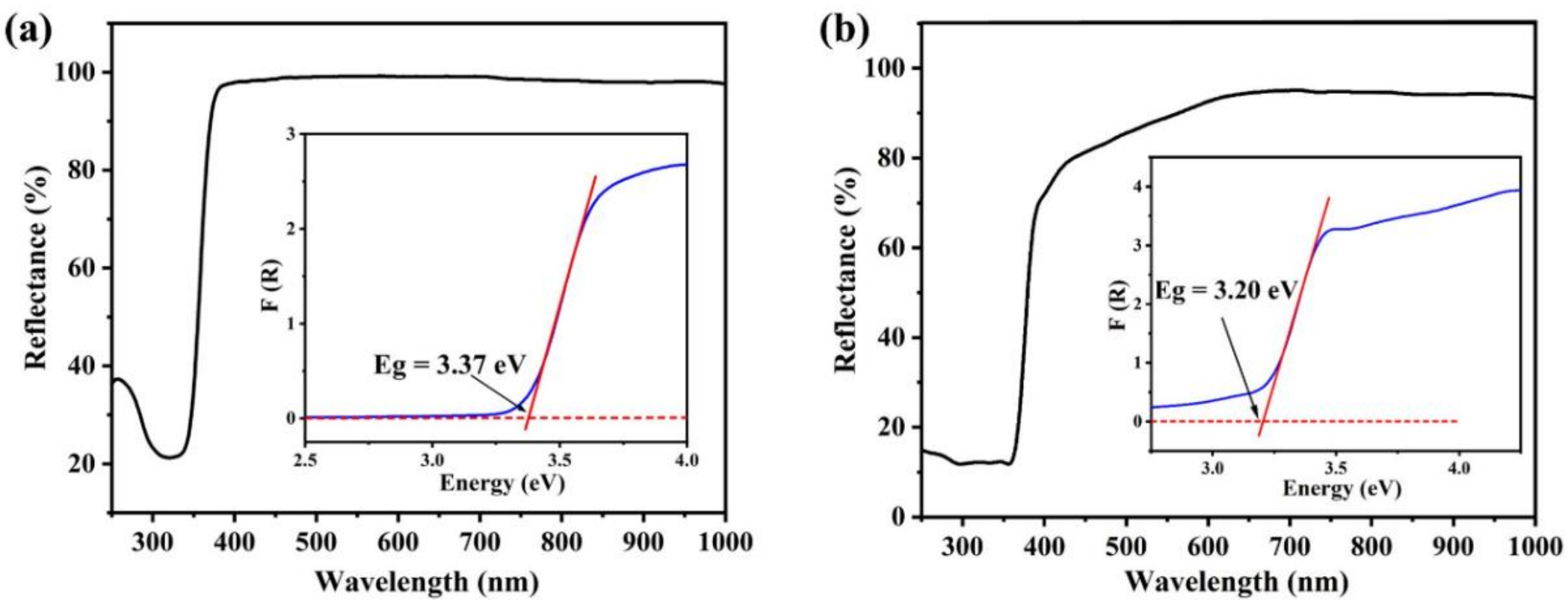

Fig. S5 UV–visible-near-infrared diffuse reflectance spectra of (a) AMPy·PO_4 and (b) AMPy·SO_4.

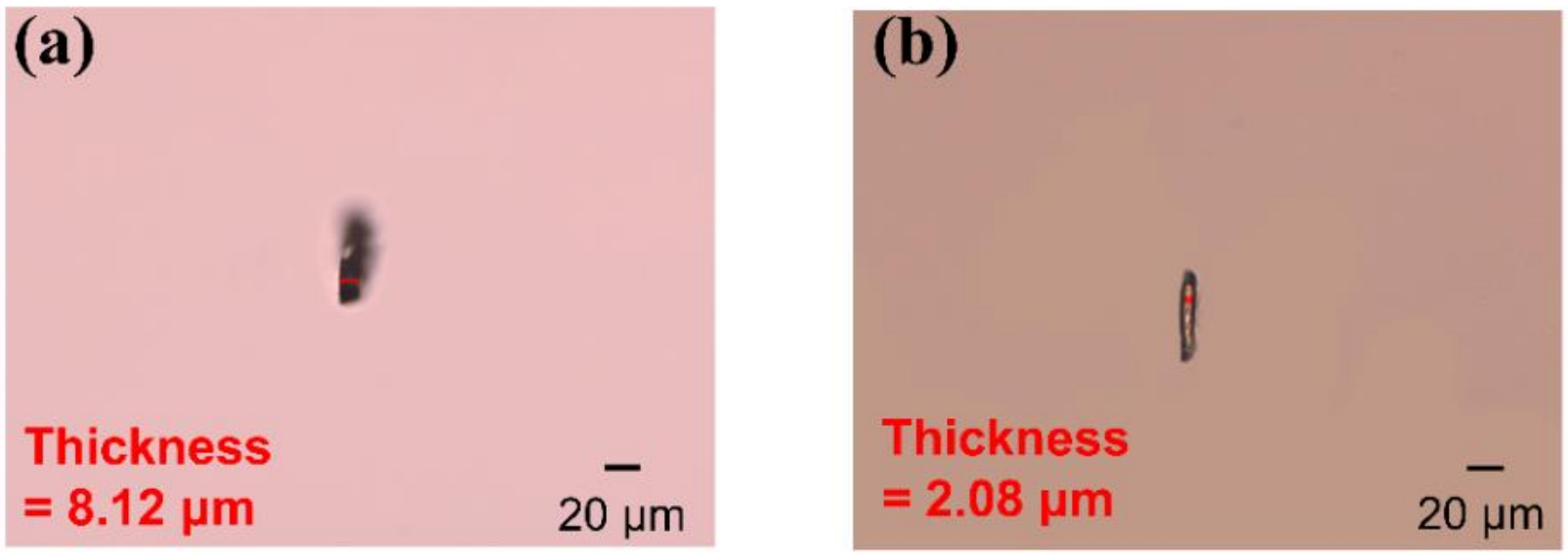

Fig. S6 Thickness of the testing crystals of for (a) AMPy PO_4 and (b) AMPy SO_4.

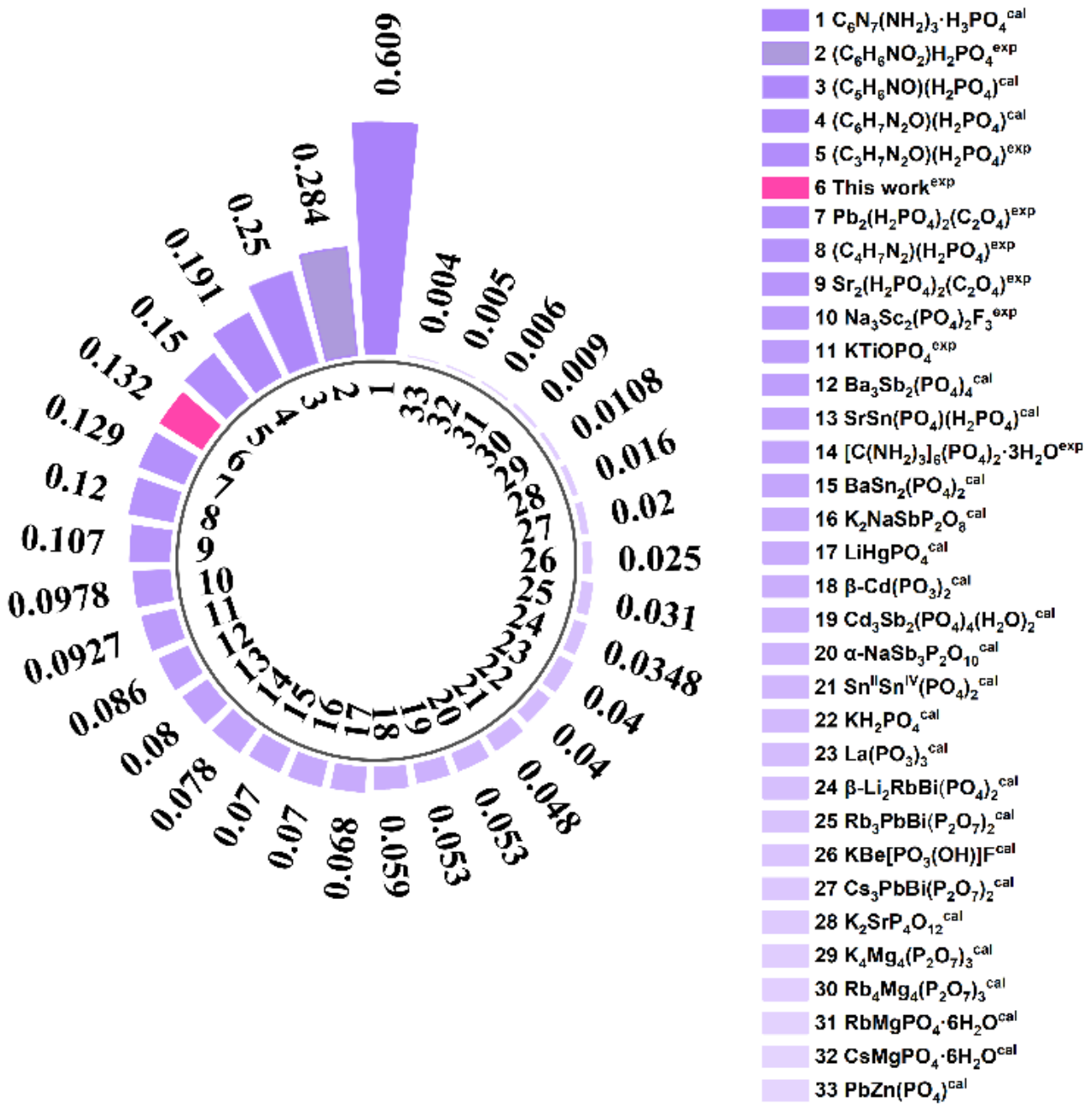

Fig. S7 Comparison of the birefringence of phosphate crystals (cal: calculated. exp: experimental).

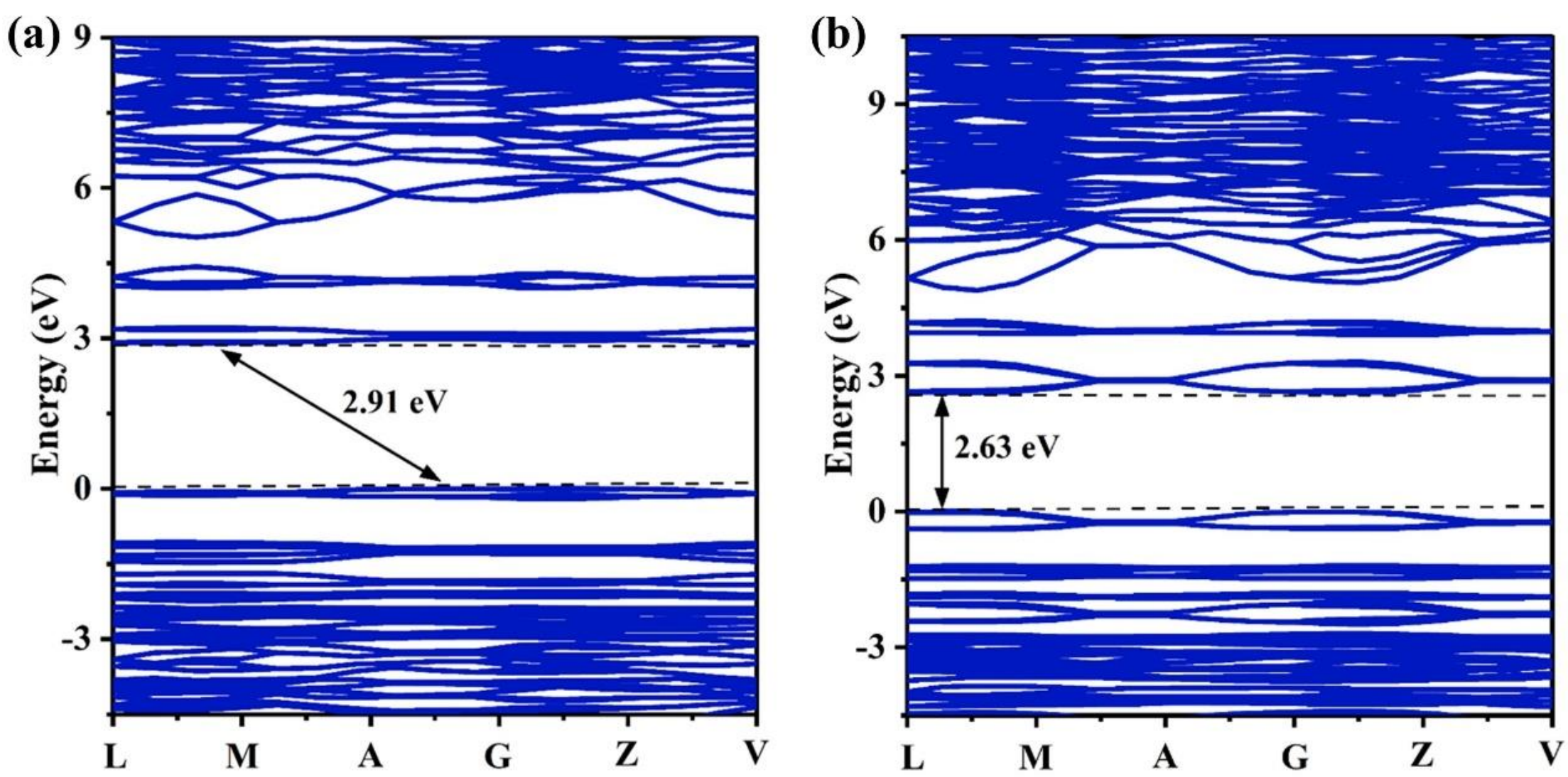

Fig. S8 Electronic band structures for (a) AMPy PO_4 and (b) AMPy SO_4.

References

1 CrysAlisPro, Rigaku Oxford Diffraction: The Woodlands, TX. 2015.

2 G. M. Sheldrick, SHELXT-Integrated Space-Group and Crystal-Structure Determination, *Acta Crystallogr. Sect. A: Found. Adv.*, 2015, **71**, 3−8.

3 G. M. Sheldrick, Crystal structure refinement with SHELXL, *Acta Crystallogr. Sect. C: Struct. Chem.*, 2015, **71**, 3−8.

4 S. J. Clark, M. D. Segall, C. J. Pickard, P. J. Hasnip, M. I. J. Probert, K. Refson and M. C. Payne, First Principles Methods Using CASTEP, *Z. Krist. Cryst. Mater*., 2005, **220**, 567−570.

5 M. C. Payne, M. P. Teter, D. C. Allan, T. A. Arias and J. D. Joannopoulos, Iterative Minimization Techniques for Ab Initio Total-Energy Calculations: Molecular Dynamics and Conjugate Gradients, *Rev. Mod. Phys.*, 1992, **64**, 1045−1097.

6 D. M. Ceperley and B. J. Alder, Ground State of the Electron Gas by a Stochastic Method, *Phys. Rev. Lett.*, 1980, **45**, 566−569.

7 J. P. Perdew and A. Zunger, Self-Interaction Correction to Density-Functional Approximations for Many-Electron Systems, *Phys. Rev. B*, 1981, **23**, 5048−5079.

8 H. J. Monkhorst and J. D. Pack, Special Points for Brillouin-Zone Integrations, *Phys. Rev. B*, 1976, **13**, 5188–5192.

9 T. Tong, W. Zhang, Z. Yang and S. Pan, Series of Crystals with Giant Optical Anisotropy: A Targeted Strategic Research, *Angew. Chem. Int. Ed.*, 2021, **60**, 1332–1338.

10 R. Wei, H. Huang, D. Yang, Y. Wang and B. Zhang, $C_6N_7(NH_2)_3{\cdot}H_3PO_4$: Strong Second Harmonic Generation and Giant Birefringence Benefiting from Amino Substitution Strategy, *Adv. Opt. Mater.*, 2024, **12**, 2401814.

11 M. B. Xu, J. Chen, H. Y. Wu, J. J. Li, N. Yu, M. F. Zhou, F. F. Mao and K. Z. Du, Designing Promising Ultraviolet (UV) Birefringent Crystals with Different Hydrogen-Bonded Phosphate Frameworks, *Inorg. Chem. Front.*, 2024, **11**, 4307−4317.

12 J. Lu, X. Liu, M. Zhao, X. B. Deng, K. X. Shi, Q. R. Wu, L. Chen and L. M. Wu, Discovery of NLO Semiorganic $(C_5H_6ON)^+(H_2PO_4)^-$: Dipole Moment Modulation and Superior Synergy in Solar-Blind UV Region, *J. Am. Chem. Soc.*, 2021, **143**, 3647−3654.

13 Q. Xia, X. Jiang, L. Qi, C. Wu, Z. Lin, Z. Huang, M. G. Humphrey, K. Tatsumi and C. Zhang, Large Optical Anisotropy in Noncentrosymmetric Phosphate with Pseudo 2D Intercalated Layer, *Inorg.*

Chem. Front., 2024, **11**, 8813−8823.

14 C. Wu, X. Jiang, Z. Wang, H. Sha, Z. Lin, Z. Huang, X. Long, M. G. Humphrey and C. Zhang, UV Solar-Blind-Region Phase-Matchable Optical Nonlinearity and Anisotropy in a π-Conjugated Cation-Containing Phosphate, *Angew. Chem. Int. Ed.*, 2021, **60**, 14718.

15 W. Wang, X. Wang, L. Xu, D. Zhang, J. Xue, S. Wang, X. Dong, L. Cao, L. Huang and G. Zou, Centrosymmetric $Rb_2Sb(C_2O_4)_{2.5}(H_2O)_3$ and Noncentrosymmetric $RbSb_2(C_2O_4)F_5$: Two Antimony (III) Oxalates as UV Optical Materials, *Inorg. Chem.*, 2023, **62**, 13148−13155.

16 F. Xu, G. Peng, C. Lin, D. Zhao, B. Li, G. Zhang, S. Yang and N. Ye, $Na_3Sc_2(PO_4)_2F_3$: Rational Design and Synthesis of an Alkali Rare-Earth Phosphate Fluoride as an Ultraviolet Nonlinear Optical Crystal with an Enlarged Birefringence, *J. Mater. Chem. C*, 2020, **8**, 4965−4972.

17 Y. V. Shaldin, R. Poprawski, The Spontaneous Birefringence and Pyroelectric Effect in $KTiOPO_4$ Crystals, *J. Phys. Chem. Solids*, 1990, **51**, 101−106.

18 X. B. Li, C. L. Hu, F. Kong and J. G. Mao, $Ba_3Sb_2(PO_4)_4$ and $Cd_3Sb_2(PO_4)_4(H_2O)_2$: Two New Antimonous Phosphates with Distinct $[Sb(PO_4)_2]$ Structure Types and Enhanced Birefringence, *Inorg. Chem.*, 2021, **60**, 1957−1964.

19 L. Deng, R. Zhang, J. Zhang, W. Xie, C. Bai, Z. Yang, X. Hou, S. Han and S. Pan, Stereochemically Active Tin (II)-Induced Enhancement of Birefringence in $Sn^{II}Sn^{IV}(PO_4)_2$ and $SrSn(PO_4)PO_2(OH)_2$, *Chem. Eur. J.*, 2023, **29**, e202300743.

20 Y. Yang, Y. Qiu, P. Gong, L. Kang, G. Song, X. Liu, J. Sun and Z. Lin, Lone-Pair Enhanced Birefringence in an Alkaline-Earth Metal Tin (II) Phosphate $BaSn_2(PO_4)_2$, *Chem. Eur. J.*, 2019, **25**, 5648−5651.

21 C. Hu, X. Cai, M. Wu, Z. Yang, J. Han and S. Pan, Lone Pair-Driven Enhancement of Birefringence in Polar Alkali Metal Antimony Phosphates, *Chem. Mater.*, 2022, **34**, 4224−4231.

22 B. L. Wu, C. L. Hu, F. F. Mao, R. L. Tang and J. G. Mao, Highly Polarizable Hg^{2+} Induced a Strong Second Harmonic Generation Signal and Large Birefringence in $LiHgPO_4$, *J. Am. Chem. Soc.*, 2019, **141**, 10188−10192.

23 J. Lv, Y. Qian, Q. Jing, X. Wang, M. H. Lee and Z. Chen, Two Metal Phosphate Nonlinear Optical Materials Simultaneously Exhibiting Ultraviolet Transparence and a Large Birefringence, *Chem. Mater.*, 2022, **34**, 5919−5927.

24 L. Wu, R. Zhang, Q. Jing, H. Huang, X. He, Z. Wang and Z. Chen, Modulating Optical Performance

by Phase Transition in a Nonlinear Optical Material β-$Li_2RbBi(PO_4)_2$, *Inorg. Chem. Front.*, 2023, **10**, 4496−4502.

25 X. Lu, Z. Chen, X. Shi, Q. Jin and M. H. Lee, Two Pyrophosphates with Large Birefringences and Second-Harmonic Responses as Ultraviolet Nonlinear Optical Materials, *Angew. Chem. Int. Ed.*, 2020, **59**, 17648−17656.

26 Y. Yang, Y. Guo, Y. G. Chen, X. Hu, X. Zhang and X. M. Zhang, Hexameric Poly-Fluoroberyllophosphate $Na_4Be_2PO_4F_5$ with Moderate Birefringence and Deep-Ultraviolet Transmission as a Potential Zero-Order-Waveplate Crystal, *Inorg. Chem. Front.*, 2022, **9**, 5469−5477.

27 Z. Bai, L. Liu, L. Zhang, Y. Huang, F. Yuan and Z. Lin, $K_2SrP_4O_{12}$: A Deep-UV Transparent Cyclophosphate as a Nonlinear Optical Crystal, *Chem. Commun.*, 2019, **55**, 8454−8457.

28 H. Yu, J. Young, H. Wu, W. Zhang, J. M. Rondinelli and P. S. Halasyamani, $M_4Mg_4(P_2O_7)_3$(M = K, Rb): Structural Engineering of Pyrophosphates for Nonlinear Optical Applications, *Chem. Mater.*, 2017, **29**, 1845−1855.

29 Y. Zhou, L. Cao, C. Lin, M. Luo, T. Yan, N. Ye and W. Cheng, $AMgPO_4{\cdot}6H_2O$ (A = Rb, Cs): Strong SHG Responses Originated from Orderly PO_4 Groups, *J. Mater. Chem. C*, 2016, **4**, 9219−9226.

30 X. B. Li, C. L. Hu, F. Kong and J. G. Mao, Structure and Optical Property Evolution in PbM (PO_4)X(M = Zn, Sn; X = Halogen): SHG Effect and Birefringence, *Inorg. Chem. Front.*, 2023, **10**, 2268−2275.

31 P. F. Li, C. L. Hu, Y. F. Li, J. G. Mao and F. Kong, $Hg_4(Te_2O_5)(SO_4)$: A Giant Birefringent Sulfate Crystal Triggered by a Highly Selective Cation, *J. Am. Chem. Soc.*, 2024, **146**, 7868−7874.

32 C. Wu, T. Wu, X. Jiang, Z. Wang, H. Sha, L. Lin, Z. Lin, Z. Huang, X. Long, M. G. Humphrey and C. Zhang, Large Second-Harmonic Response and Giant Birefringence of $CeF_2(SO_4)$ Induced by Highly Polarizable Polyhedra, *J. Am. Chem. Soc.*, 2021, **143**, 4138−4142.

33 T. Wu, X. Jiang, Y. Zhang, Z. Wang, H. Sha, C. Wu, Z. Lin, Z. Huang, X. Long, M. G. Humphrey and C. Zhang, From $CeF_2(SO_4){\cdot}H_2O$ to $Ce(IO_3)_2(SO_4)$: Defluorinated Homovalent Substitution for Strong Second-Harmonic-Generation Effect and Sufficient Birefringence, *Chem. Mater.*, 2021, **33**, 9317−9325.

34 L. Zhang, X. Zhang, F. Liang, Z. Hu and Y. Wu, Rational Design of Noncentrosymmetric Organic-Inorganic Hybrids with a π-Conjugated Pyridium-Type Cation for High Nonlinear-Optical Performance, *Inorg. Chem.*, 2023, **62**, 14518−14522.

35 H. X. Tang, Y. X. Zhang, C. Zhuo, R. B. Fu, H. Lin, Z. J. Ma and X. T. Wu, A Niobium Oxyiodate Sulfate with a Strong Second-Harmonic-Generation Response Built by Rational Multi-Component Design, *Angew. Chem. Int. Ed.*, 2019, **58**, 3824−3828.

36 P. F. Li, C. L. Hu, Y. P. Gong, F. Kong and J. G. Mao, $Hg_3(Te_3O_8)(SO_4)$: A New Sulfate Tellurite with a Novel Structure and Large Birefringence Explored from d^{10} Metal Compounds, *Chem. Commun.*, 2021, **57**, 7039−7042.

37 Y. Lan, J. Ren, P. Zhang, X. Dong, L. Huang, L. Cao, D. Gao and G. Zou, $ASb(SO_4)_2$(A = Rb, Cs): Two Short-Wave UV Antimony Sulfates Exhibiting Large Birefringence, *Chin. Chem. Lett.*, 2024, **35**, 108652.

38 Y. L. Li, M. Y. Ji, C. L. Hu, J. Chen, B. X. Li, Y. Lin and J. G. Mao, Explorations of New SHG Materials in Mercury Iodate Sulfate System, *Chem. Eur. J.*, 2022, **28**, e202200001.

39 Y. Lan, H. Luo, L. Wang, L. Huang, L. Cao, X. Dong and G. Zou, Two Short-Wave UV Antimony (III) Sulfates Exhibiting Large Birefringence, *Inorg. Chem.*, 2024, **63**, 2814−2820.

40 P. F. Li, C. L. Hu, F. Kong, S. M. Ying and J. G. Mao, $Y_2(Te_4O_{10})(SO_4)$: A New Sulfate Tellurite with a Unique Te_4O_{10} Polyanion and Large Birefringence, *Inorg. Chem. Front.*, 2021, **8**, 164−172.

41 Q. Wei, K. Wang, C. He, L. Wei, X. F. Li, S. Zhang, X. T. An, J. H. Li and G. M. Wang, Linear and Nonlinear Optical Properties of Centrosymmetric $Sb_4O_5SO_4$ and Noncentrosymmetric $Sb_4O_4(SO_4)(OH)_2$ Induced by Lone Pair Stereoactivity, *Inorg. Chem.*, 2021, **60**, 11648−11654.

42 X. Dong, Z. Zhang, L. Huang and G. Zou, $[C(NH_2)_3]BiCl_2SO_4$: An Excellent Birefringent Material Obtained by Multifunctional Group Synergy, *Inorg. Chem. Front.*, 2022, **9**, 5572−5578.

43 F. He, Y. Ge, X. Zhao, J. He, L. Huang, D. Gao, J. Bi, X. Wang and G. Zou, Two-Stage Evolution from Phosphate to Sulfate of New KTP-Type Family Members as UV Nonlinear Optical Materials through Chemical Cosubstitution-Oriented Design, *Dalton Trans.*, 2020, **49**, 5276−5282.

44 X. F. Li, K. Wang, C. He, J. H. Li, X. T. An, J. Pan, Q. Wei, G. M. Wang and G. Y. Yang, $Sb_4O_3(TeO_3)_2(HSO_4)(OH)$: An Antimony Tellurite Sulfate Exhibiting Large Optical Anisotropy Activated by Lone Pair Stereoactivity, *Inorg. Chem.*, 2023, **62**, 7123−7129.

45 X. Hao, S. Xie, R. Wang, C. Lin, L. Wu, G. Peng, T. Yan, N. Ye and M. Luo, $(CN_4H_7)_2SO_4{\cdot}H_2O$: High-Performance Metal-Free Ultraviolet Birefringent Crystal with KBBF-Like Configuration, *Inorg. Chem. Front.*, 2025, **12**, 5335−5343.

46 Z. Bai and K. M. Ok, Dramatically Improved Optical Anisotropy by Realizing Stereochemically

Active Lone Pairs in a Sulfate System, $K_2SO_4{\cdot}HIO_3$, *Inorg. Chem. Front.*, 2023, **10**, 1919−1925.

47 H. Fu, X. Zhang, P. Liu, B. Li, B. Wu, Y. Tao, Q. Lu, Y. Li, J. Huang, F. Zhang, T. He, Z. Chen, H. Wang, C. Su, H. Y. Zang, X. Yu and X. Li, A Chiral Sodium Lanthanum Sulfate for Second-Order Nonlinear Optics and Proton Conduction, *Inorg. Chem. Front.*, 2024, **11**, 7026−7033.

48 X. Dong, L. Huang, H. Zeng, Z. Lin, K. M. Ok and G. Zou, High-Performance Sulfate Optical Materials Exhibiting Giant Second Harmonic Generation and Large Birefringence, *Angew. Chem. Int. Ed.*, 2022, **61**, e202116790.

49 J. Ren, Y. Chen, L. Ren, Y. Zhou, X. Dong, D. Gao, L. Huang, L. Cao and N. Ye, $Hg_3(SeO_3)_2(SO_4)$: A UV Nonlinear Optical Mercury Selenite Sulfate Constructed by Neat $[Hg_6O_8(SeO_3)_4]_\infty$ Layers and SO_4 Tetrahedra, *Inorg. Chem.*, 2023, **62**, 9130−9138.

50 Q. Wang, L. Wang, X. Zhao, L. Huang, D. Gao, J. Bi, X. Wang and G. Zou, Centrosymmetric $K_2SO_4{\cdot}(SbF_3)_2$ and Noncentrosymmetric $Rb_2SO_4{\cdot}(SbF_3)_2$ Resulting from Cooperative Effects of Lone Pair and Cation Size, *Inorg. Chem. Front.*, 2019, **6**, 3125−3132.

51 F. He, Y. Deng, X. Zhao, L. Huang, D. Gao, J. Bi, X. Wang and G. Zou, $RbSbSO_4Cl_2$: An Excellent Sulfate Nonlinear Optical Material Generated Due to the Synergistic Effect of Three Asymmetric Chromophores, *J. Mater. Chem. C*, 2019, **7**, 5748−5754.

52 K. Sudhakar, S. Nandhini, S. Muniyappan, T. Arumanayagam, P. Vivek and P. Murugakoothan, Synthesis, Crystal Growth, Optical, Thermal, and Mechanical Properties of a Nonlinear Optical Single Crystal: Ammonium Sulfate Hydrogen Sulphamate (ASHS), *Appl. Phys. A*, 2018, **124**, 334.

53 Y. Li, C. Hu, J. Chen and J. Mao, Two Bismuth Iodate Sulfates with Enhanced Optical Anisotropy, *Dalton Trans.*, 2021, **50**, 16139−16146.

54 Y. Shang, H. Sha, Z. Wang, R. Su, C. He, X. Yang and X. Long, The Birefringence Modulation in Short-Wave Ultraviolet Sulfates with Functional π-Conjugated Cations and Polymerized Heteroleptic Tetrahedral Anions, *Adv. Opt. Mater.*, 2024, **12**, 2302844.
